# Discordant Impact of HLA on Viral Replicative Capacity and Disease Progression in Pediatric and Adult HIV Infection

**DOI:** 10.1371/journal.ppat.1004954

**Published:** 2015-06-15

**Authors:** Emily Adland, Paolo Paioni, Christina Thobakgale, Leana Laker, Luisa Mori, Maximilian Muenchhoff, Anna Csala, Margaret Clapson, Jacquie Flynn, Vas Novelli, Jacob Hurst, Vanessa Naidoo, Roger Shapiro, Kuan-Hsiang Gary Huang, John Frater, Andrew Prendergast, Julia G. Prado, Thumbi Ndung’u, Bruce D. Walker, Mary Carrington, Pieter Jooste, Philip J. R. Goulder

**Affiliations:** 1 Department of Paediatrics, University of Oxford, Peter Medawar Building for Pathogen Research, Oxford, United Kingdom; 2 HIV Pathogenesis Programme, Doris Duke Medical Research Institute, University of KwaZulu-Natal, Durban, South Africa; 3 Paediatric Department, Kimberley Hospital, Northern Cape, South Africa; 4 Department of Paediatric Infectious Diseases, Great Ormond St Hospital for Children, London, United Kingdom; 5 The Institute for Emerging Infections, The Oxford Martin School, University of Oxford, Oxford, United Kingdom; 6 Nuffield Department of Medicine, University of Oxford, Peter Medawar Building for Pathogen Research, Oxford, United Kingdom; 7 Oxford National Institute of Health Research, Biomedical Research Centre, Oxford, United Kingdom; 8 Botswana Harvard AIDS Institute Partnership, Gaborone, Botswana; 9 Centre for Paediatrics, Blizard Institute, Queen Mary University of London, London, United Kingdom; 10 Zvitambo Institute for Maternal and Child Health Research, Harare, Zimbabwe; 11 AIDS Research Institute IrsiCaixa, Institut d'Investigació en Ciències de la Salut Germans Trias i Pujol, Badalona, Barcelona, Spain; 12 The Ragon Institute of Massachusetts General Hospital (MGH), Massachusetts Institute of Technology (MIT), and Harvard University, Boston, Massachusetts, United States of America; 13 KwaZulu-Natal Research Institute for Tuberculosis and HIV, University of KwaZulu-Natal, Durban, South Africa; 14 Max Planck Institute for Infection Biology, Berlin, Germany; 15 Center for Cancer Research, National Cancer Institute, Frederick, Maryland, United States of America; Vaccine Research Center, UNITED STATES

## Abstract

HLA class I polymorphism has a major influence on adult HIV disease progression. An important mechanism mediating this effect is the impact on viral replicative capacity (VRC) of the escape mutations selected in response to HLA-restricted CD8+ T-cell responses. Factors that contribute to slow progression in pediatric HIV infection are less well understood. We here investigate the relationship between VRC and disease progression in pediatric infection, and the effect of HLA on VRC and on disease outcome in adult and pediatric infection. Studying a South African cohort of >350 ART-naïve, HIV-infected children and their mothers, we first observed that pediatric disease progression is significantly correlated with VRC. As expected, VRCs in mother-child pairs were strongly correlated (p = 0.004). The impact of the protective HLA alleles, HLA-B*57, HLA-B*58:01 and HLA-B*81:01, resulted in significantly lower VRCs in adults (p<0.0001), but not in children. Similarly, in adults, but not in children, VRCs were significantly higher in subjects expressing the disease-susceptible alleles HLA-B*18:01/45:01/58:02 (p = 0.007). Irrespective of the subject, VRCs were strongly correlated with the number of Gag CD8+ T-cell escape mutants driven by HLA-B*57/58:01/81:01 present in each virus (p = 0.0002). In contrast to the impact of VRC common to progression in adults and children, the HLA effects on disease outcome, that are substantial in adults, are small and statistically insignificant in infected children. These data further highlight the important role that VRC plays both in adult and pediatric progression, and demonstrate that HLA-independent factors, yet to be fully defined, are predominantly responsible for pediatric non-progression.

## Author Summary

HLA plays a central role in determining disease outcome in adult HIV infection. A principal mechanism by which this HLA effect is mediated is via viral replicative capacity (VRC), protective HLA alleles such as HLA-B*57 driving the selection of viral escape mutants that reduce VRC. The factors contributing to the diverse disease progression rates observed in infected children, however, remain uncertain. We here address the role of HLA and VRC in pediatric disease progression in a large cohort in Kimberley, South Africa. The findings highlight the consistent and important role of VRC in both adult and pediatric progression. However, the impact of key HLA molecules in shaping disease outcome in adult infection is notably absent in pediatric infection. Further studies of pediatric infection therefore provide the potential to gain critical new insights into HLA-independent mechanisms of HIV disease non-progression that predominate in HIV-infected but healthy, ART-naive children. Understanding these mechanisms remains of direct relevance to the development of future interventions to minimize HIV disease.

## Introduction

HIV-infected children typically progress more rapidly to AIDS than adults [1]. Without treatment, approximately 50% of HIV-1 infected children develop AIDS within a year and 50% will die before their second birthday in sub-Saharan Africa [1]. However, a minority of perinatally infected children remains asymptomatic through childhood despite being antiretroviral therapy (ART) naive. The reasons for this variation in pediatric disease progression remain largely unknown. Although pediatric HIV infections have accounted for only approximately 10% of those arising in the global epidemic (http://www.avert.org/global-hiv-aids-epidemic.htm), they provide the potential to understand new mechanisms by which HIV disease can be avoided following infection. This remains an important goal of HIV research, with direct relevance to vaccine design.

The central role of HLA class I molecules in control of adult HIV infection is underlined by several genome-wide association studies [2–4]. These are consistent with earlier work showing characteristic rates of disease progression, depending upon the particular HLA molecules expressed [5]. In African populations, HLA-B*57, HLA-B*58:01 and HLA-B*81:01 are the class I molecules most consistently associated with slow progression to HIV disease and HLA-B*18:01, HLA-B*45:01 and HLA-B*58:02 the HLA alleles most strongly associated with high viral load and rapid disease progression [2, 6–11].

Several distinct mechanisms are likely to contribute to the immune control or lack of immune control of HIV mediated by these particular HLA-B alleles [5]. One important mechanism is believed to be the impact of selection pressure imposed on HIV by Gag-specific CD8+ T cell responses. Viral escape mutants selected to evade the Gag-specific responses restricted by protective HLA alleles tend to reduce viral replicative capacity [12–20]. Although in some cases compensatory mutations have been selected that almost cancel out the fitness cost of the escape mutant [21], in most cases the compensatory mutations only partially recover the reduction in viral replicative capacity resulting from selection of the escape mutant [14,19,20,22,23]. In contrast with protective HLA molecules, those that mediate lack of immune control typically restrict dominant epitopes outside of Gag, in proteins such as Env and Nef [24], and escape mutants are either not selected by these CTL responses or they have no discernible effect on viral replicative capacity [24–26]. Thus the mechanism of HLA-mediated immune control or lack of control is strongly linked to the impact that the relevant escape mutants have on viral replicative capacity.

The role of HLA and the impact of viral replicative capacity on disease progression has been less well studied in pediatric HIV infection. Although CD8+ T cell responses against HIV are detectable in the first days of life [27,28], a viral setpoint is not established for years [29], unlike adult infection where viral load reaches a setpoint 6 weeks after infection [1]. The evidence that HLA class I plays an important role in immune control of HIV in infected children is mixed and somewhat contradictory [30–36]. In the largest of these studies, which followed 61 HIV-infected mother-child pairs from birth, there was no overall impact of HLA on rate of progression in children, but if either the child or mother carried a protective HLA allele (HLA-B*57/58:01/81:01), progression in the child was significantly delayed, provided that the protective allele was not shared by mother and child. This supported earlier work [30,31], and was reinforced by subsequent studies in adults showing that disease progression in the recipient is related to the viral replicative capacity of the transmitted virus [37–40]. Nonetheless, findings from a smaller study of 11 Jamaican children suggested that infected children did not benefit from having an HLA-B*57-positive mother [34]. However, in none of these studies were direct measurements of viral replicative capacity made in infected children and their transmitting mother, with the exception of one study [35] that measured VRC in 8 children only, and therefore was unable to examine the impact of HLA on VRC in pediatric infection.

To better define the relationship between viral replicative capacity (VRC), HLA expression, and disease outcome in pediatric infection, we here studied a cohort of >350 HIV infected children in South Africa. These studies are the first to measure viral replicative capacity both in infected children and in their transmitting mothers, enabling the impact of transmitted virus and maternal HLA type on pediatric VRC and disease outcome to be directly assessed. In addition, these investigations are the first to be undertaken in sufficient numbers of study subjects to analyze with statistical rigor the impact of the VRC and HLA in both mother and child on pediatric disease outcome. We aimed, first, to determine the relationship between viral replicative capacity and disease progression in pediatric infection; second, to assess the impact of maternal HLA and maternal VRC on VRC in the child; third, to compare the impact of both protective and disease-susceptible HLA alleles, respectively, on the VRC of viruses in adults and children; and, finally, to compare the impact of protective and disease-susceptible HLA on disease outcome adults and children.

## Materials and Methods

### Patients and samples

We studied viral replicative capacity in a total of 47 treatment naïve mothers and 84 treatment naïve children with chronic HIV-1 C-clade infection from Kimberley, South Africa. The mean absolute CD4 count of the mothers was 358/mm^3^ (IQR 235-449/mm^3^) and the median viral load 65,000 copies/ml (IQR 17,797–264,330). Of the 84 children studied, 55 were defined as slow progressors based on failure to meet 2010 WHO criteria to initiate ART. These criteria may be summarised thus: (i) age (all those diagnosed at <1yr are recommended to initiate ART irrespective of CD4 count) (ii) CD4 criteria to initiate ART: a CD4%<25% in children aged 1–4yrs or an absolute CD4<350/mm^3^ in children aged ≥5yrs; (iii) clinical criteria to start ART (WHO clinical disease stage 3 or 4). The mean age of the slow progressors (n = 55) was 7.5yrs (IQR 66–112 months), mean CD4% 26% (IQR 21–29%), mean absolute CD4 count 642/ mm^3^ (IQR 422-824/ mm^3^) and median viral load 30,000 copies/ml (IQR 6,800–79,000). Rapid progressor children (n = 29) were defined as those who had reached a CD4%<25% by 24 months of age. The mean age of the rapid progressors was 14 months (IQR 8–23months), mean CD4% 16% (IQR 8–20%), mean absolute CD4 count 554/mm^3^ (IQR 226-831/mm^3^) and median viral load 1,100,000 copies/ml (IQR 280,000–5,650,000). [For comparison with published data for HIV-uninfected children [41] aged 1–2yrs, at this age the normal range (10^th^-90^th^ centiles) of absolute CD4 count is 1300-3400/mm^3^ and of CD4% is 32–51%].

The HLA typed adult cohort analyzed to compare the impact of protective and disease-susceptible alleles in infected adults versus children, comprised 1,211 ART-naïve subjects with chronic clade C HIV infection from Durban, South Africa. This cohort has previously been well-described [8,24]. The median CD4 count of the cohort was 376/mm^3^, (IQR 240–518/mm^3^). The median viral load was 38,200 copies/ml (IQR 7,209–156,000). The 361 HLA typed, HIV-infected children analyzed comprised 310 children from Kimberley, South Africa; 44 children from Durban, South Africa, including 28 children from a previously described smaller cohort of children [33,42] who were followed from birth; and 7 Southern African children being followed in UK. The Durban cohort included 17 ART-naïve children followed from birth, of whom 5 expressed one of the protective HLA alleles HLA-B*57/58:01/81:01. Among the pediatric ‘rapid progressors’ (n = 98), defined using the criteria above (CD4%<25% by 24 months of age), the median age immediately prior to ART initiation was 6m, (IQR 3–9m), median absolute CD4 count 584/mm^3^ (IQR 321–952/mm^3^), median CD4% 13% (IQR 9.5–15%), and median viral load 1.1m copies/ml (IQR 0.35–3.0m); and ‘slow progressors’ (n = 263): ART-naïve children aged ≥5yrs (mean 7.8yrs, IQR 5.1–10.0yrs), median absolute CD4 count 845/mm^3^ (IQR 470–1084/mm^3^), median CD4% 26% (IQR 19–31%), median viral load 34,000 (IQR 6,630–110,000).

Viral load measurement was undertaken using the BioMérieux NucliSens Version 2.0 Easy Q/ Easy Mag (NucliSens v2.0) assay (dynamic range 20–10m) prior to 2010 and using the COBAS AmpliPrep/COBAS TaqMan HIV-1 Test version 2.0 by Roche (CAP/CTM v2.0) (dynamic range 20–10m) subsequent to 2010. In the case of child P1, plasma was diluted 10 fold to determine the viral load above the 10m upper limit of the assay. CD4+ T cell counts were measured by flow cytometry.

Informed consent was provided for participation of the subjects in the study. Ethics approval was given by the University of the Free State Ethics Committee, Bloemfontein, the Biomedical research Ethics Committee, University of KwaZulu-Natal, Durban, and the Oxford Research Ethics Committee.

### HLA typing

Samples from study subjects were HLA-A,-B, and–C Sequence Based Typed in the CLIA/ASHI accredited laboratory of William Hildebrand, PhD, D (ABHI) at the University of Oklahoma Health Sciences Center using a locus specific PCR amplification strategy and a heterozygous DNA sequencing methodology for exon 2 and 3 of the class I PCR amplicon.

Relevant ambiguities [43] were resolved by homozygous sequencing. DNA sequence analysis and HLA allele assignment were performed with the software Assign-SBT v3.5.1 from Conexio Genomics.

### Viral RNA isolation and nested RT-PCR amplification of gag-protease from plasma

Viral RNA was isolated from plasma by use of a QIAamp Viral RNA Mini Kit from Qiagen. The Gag-Protease region was amplified by reverse transcription (RT)-PCR from plasma HIV-1 RNA using Superscript III One-Step Reverse Transcriptase kit (Invitrogen) and the following Gag-protease-specific primers: 5’ CAC TGC TTA AGC CTC AAT AAA GCT TGC C 3’ (HXB2 nucleotides 512 to 539) and 5’ TTT AAC CCT GCT GGG TGT GGT ATT CCT 3’ (nucleotides 2851 to 2825). Second round PCR was performed using 100-mer primers that completely matched the pNL4-3 sequence using Takara EX Taq DNA polymerase, Hot Start version (Takara Bio Inc., Shiga, Japan). One hundred microliters of reaction mixture was composed of 10ul of 10x EX Taq buffer, 4ul of deoxynucleoside triphosphate mix (2.5 mM each), 6ul of 10uM forward primer (GAC TCG GCT TGC TGA AGC GCG CAC GGC AAG AGG CGA GGG GCG ACT GGT GAG TAC GCC AAA AAT TTT GAC TAG CGG AGG CTA GAA GGA GAG AGA TGG G, 695 to 794) and reverse primer (GGC CCA ATT TTT GAA ATT TTT CCT TCC TTT TCC ATT TCT GTA CAA ATT TCT ACT AAT GCT TTT ATT TTT TCT GTC AAT GGC CAT TGT TTA ACT TTT G, 2646 to 2547), 0.5ul of enzyme, and 2ul of first round PCR product and DNase-RNase-free water. Thermal cycler conditions were as follows: 94°C for 2 min, followed by 40 cycles of 94°C for 30 s, 60°C for 30 s, and 72°C for 2 min and then followed by 7 min at 72°C. PCR products were purified using a QIAquick PCR purification kit (Qiagen, UK) according to manufacturer’s instructions.

### Generation of recombinant Gag-Protease viruses

A deleted version of pNL4-3 was constructed [17] that lacks the entire Gag and Protease region (Stratagene Quick-Change XL kit) replacing this region with a BstE II (New England Biolabs) restriction site at the 5’ end of Gag and the 3’ end of protease. To generate recombinant viruses, 10ug of BstEII-linearized plasmid plus 50ul of the second-round amplicon (approximately 2.5ug) were mixed with 2 x 10^6^ cells of a Tat-driven green fluorescent protein (GFP) reporter T cell line [44] (GXR 25 cells) in 800ul of R10 medium (RPMI 1640 medium containing 10% fetal calf serum, 2 mM L-glutamine, 100 units/ml penicillin and 100ug/ml streptomycin) and transfected by electroporation using a Bio-Rad GenePulser II instrument (300 V and 500 uF). Following transfection, cells were rested for 45 min at room temperature, transferred to T25 culture flasks in 5ml warm R10 and fed with 5ml R10 on day 4. GFP expression was monitored by flow cytometry (FACS Calibur; BD Biosciences), and once GFP expression reached >30% among viable cells supernatants containing the recombinant viruses were harvested and aliquots stored at -80°C.

### Replication capacity assays

The replication capacity of each chimera is determined by infection of GXR cells at a low multiplicity of infection (MOI) of 0.003. The mean slope of exponential growth from day 2 to day 7 was calculated using the semi log method in Excel. This was divided by the slope of growth of the wild-type NL4-3 control included in each assay to generate a normalized measure of replication capacity. Replication assays were performed in triplicate, and results were averaged. These VRC determinations were undertaken entirely blinded to the identity of the study subject.

### Viral sequencing and phylogenetic analysis

Population sequencing was undertaken using the Big Dye ready reaction terminator mix (V3) (Department of Zoology, University of Oxford). Sequence data were analysed using Sequencher v.4.8 (Gene Codes Corporation). Nucleotides for each gene were aligned manually in Se-Al v.2.0a11. Maximum-Likelihood phylogenetic trees were generated using PHYm131 (http://www.hiv.lanl.gov) and visualised using Figtree v.1.2.2 (http://tree.bio.ed.ac.uk/software/figtee/).

### Interferon-gamma elispot and ICS assays

As previously described, [7,28,33] interferon gamma elispot assays were carried out using a panel of overlapping peptides (18mers with a 10 amino acid overlap) that had been synthesized based on the clade C consensus sequence; 66 peptides spanned the Gag protein sequence. Specific responses of >100 spot forming cells (SFC)/million peripheral blood mononuclear cells (PBMC) were defined as significant. In addition, defined optimal peptides were also tested according to the corresponding HLA type of the subject. The ICS assays presented were performed as previously described [30] using the pool of 66 overlapping Gag peptides. Quadrant boundaries for IFN-γ staining were established by exclusion of 99.9% of control CD8+ T cells.

### Statistical analysis

The VRCs of rapid progressor and slow progressor children were compared using the Student’s t test. These data were normally distributed as determined by the D’Agostino and Pearson omnibus normality test. The Mann-Whitney U Test was used to compare differences in VRC resulting from protective or disease-susceptible HLA allele expression as these data were not normally distributed (D’Agostino and Pearson omnibus normality test). The relationship between maternal VRC and child VRC and CD4 and VRC were assessed using the Spearman’s correlation. The relationship between VRC and number of selected Gag mutations was also assessed by determination of the Spearman rank correlation coefficient between the two variables. The selected mutants comprised [11,15,45,46]: A146X and I147X within the HLA-B*57 restricted epitope ISPRTLNAW (ISW9, Gag 147–155); A163X and S165X within the HLA-B*57:03-restricted epitope KAFSPEVIPMF (KF11, Gag 162–172); E177X, Q182X and T186S within the HLA-B*81:01-restricted epitope TPQDLNTML (TL9, Gag 180–188); T242X and I247X within the HLA-B*57/58:01-restricted epitope TSTLQEQIAW (TW10, Gag 240–249); T310X within the HLA-B*58:01-restricted epitope QATQDVKNW, (QW9, Gag 308–316). In the analyses of the impact of HLA on CD4 count and viral load in pediatric study subjects, we undertook power calculations based on previously published adult data [8,11] via bootstrap experiments where we sampled with replacement from the adult data. For example, to test the difference in viral loads between the subjects expressing Protective HLA alleles and those expressing Disease-susceptible patients, for each study group sample size, 1000 experiments were undertaken to determine the proportion where the difference in viral loads between Protective and Disease-susceptible groups remained significant at p<0.05. The number in each group required to provide 80% power to show this significance difference between the groups was a group size of 27; having as many as 46 in each group provided 97% power. These calculations demonstrate that we had ample statistical power to demonstrate whether there was an HLA effect in children that was equivalent to that observed in adults.

## Results

### Lower viral replicative capacity associated with slow progression in children

Recombinant viruses were generated from 43 mother-child pairs, and from an additional 41 children for whom the mother was unavailable for study and a further 4 mothers for whom the child was unavailable for study. Fifty-five of these children were defined as slow-progressor (ART-naïve, absolute CD4 count >350/mm^3^, age ≥5yrs, did not meet 2010 WHO criteria to commence ART) and 29 were defined as rapid-progressor (CD4 ≤25% by 24 months of age). Chimeric viruses generated by co-transfection of the amplified patient-derived gag-protease genes with the linearized pNL4-3Δgag-protease were used to infect a GFP reporter cell line as previously described [18,19,44,45,47–51]. Several previous studies adopting this methodology [18,45,47,48] have demonstrated 99–100% amino acid sequence identity between plasma virus and the gag-pro-chimeric viruses, after exclusion of codons containing mixed bases. The same comparison was undertaken here in a subset of the study subjects (n = 33), confirming amino acid identity between plasma and gag-pro-chimeric viruses sequence at a median of 99.4% residues (range IQR 99–100%) (S1A and S1B Fig). Thus the gag-pro chimeric viruses that were generated are indeed representative of the plasma virus in each study subject.

**Fig 1:**
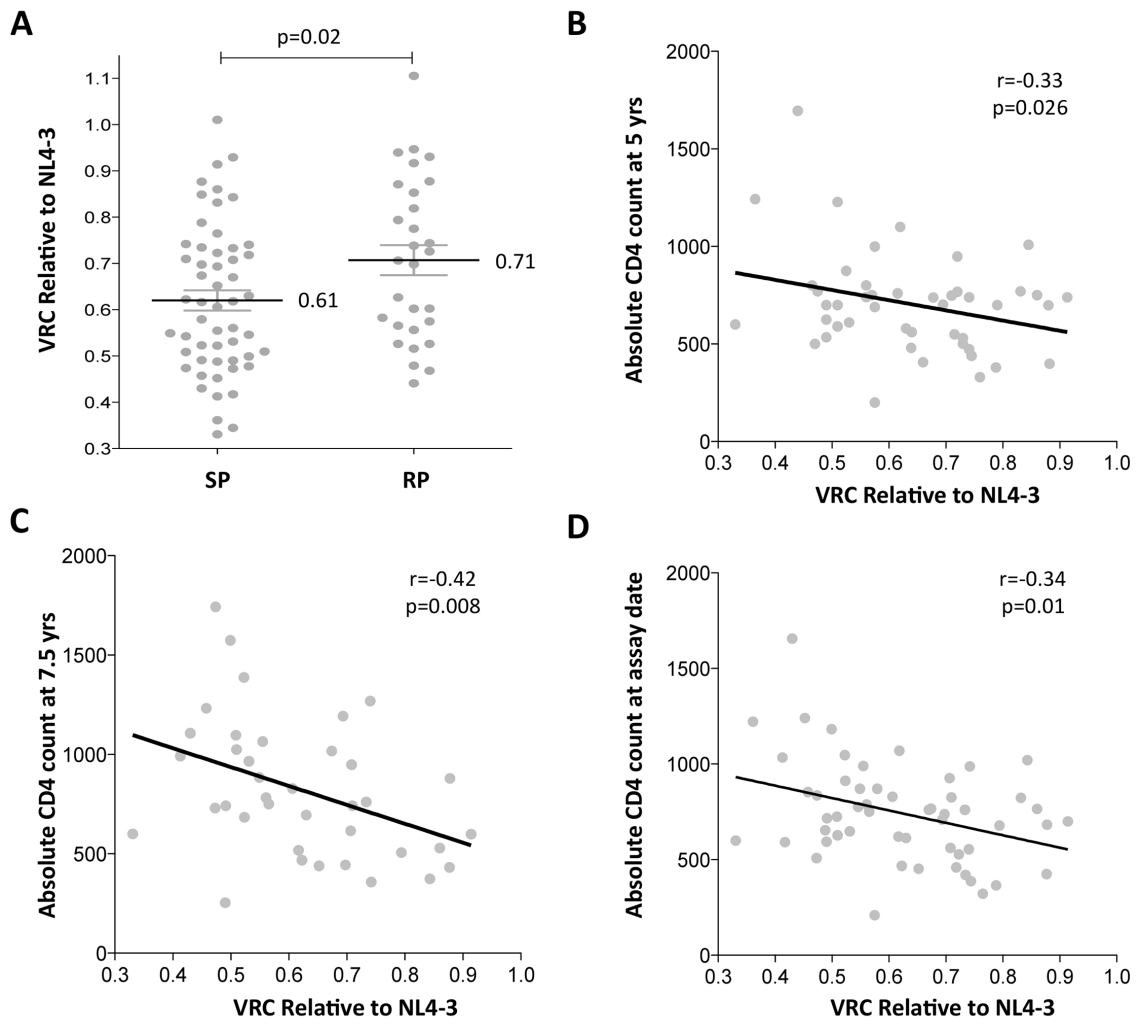
Lower viral replicative capacity is associated with slow progression in children. A. Replication capacities, normalized to NL4-3 comparator virus, were significantly lower in slow progressor children, median 0.61 (n = 55) compared to rapid progressor children, median 0.71 (n = 29) (p = 0.02). B. VRC correlation with decreasing absolute CD4 count in slow-progressor children at 5 years of age (n = 46). C. VRC correlation with decreasing absolute CD4 count in slow-progressor children at 5 years of age (n = 38). D. VRC correlation with decreasing absolute CD4 count in slow-progressor children at the age of assay date (n = 55).

Gag-protease chimeric viruses derived from slow-progressor children displayed significantly reduced replication capacity to those derived from rapid-progressors (p = 0.02; Student’s t test; Fig 1A). Furthermore, VRC correlated significantly with decreasing absolute CD4 count in the slow-progressor children at 5 yrs of age (r = -0.33 p = 0.026, Fig 1B) and at 7.5 yrs of age (r = -0.42, p = 0.008, Fig 1C). The plasma samples from which the chimeric viruses were generated were obtained from slow progressors aged a mean of 7.5 years, and thus the stronger correlation with CD4 count at 7.5 years than with CD4 count at 5yrs is consistent with this. Similarly, the measured VRC also correlated with the CD4 count exactly coincident with sample obtained for generation of the chimeric viruses from which VRC was determined (r = -0.34, p = 0.01; Fig 1D). Together these data suggest that VRC influences disease progression in pediatric infection, with CD4 count being closely associated with disease progression in HIV-infected children.

Of note, in contrast to HIV-infected adults, viral load and absolute CD4 are not significantly correlated, except in older children as they approach adolescence. Here, in the slow progressor children aged 5yrs, the correlation between VL and CD4 is weak (r = -0.25, p = 0.32) and in the 7.5yo children, the correlation approaches significance (r = -0.31; p = 0.09) (S2 Fig). For this reason, there is no correlation observed between VRC and viral load in the slow progressor children (S2 Fig). In contrast to absolute CD4 count, viral load is a poor marker of disease progression in children and is not part of WHO criteria for ART initiation in children.

### Impact of the maternally transmitted virus

**Fig 2:**
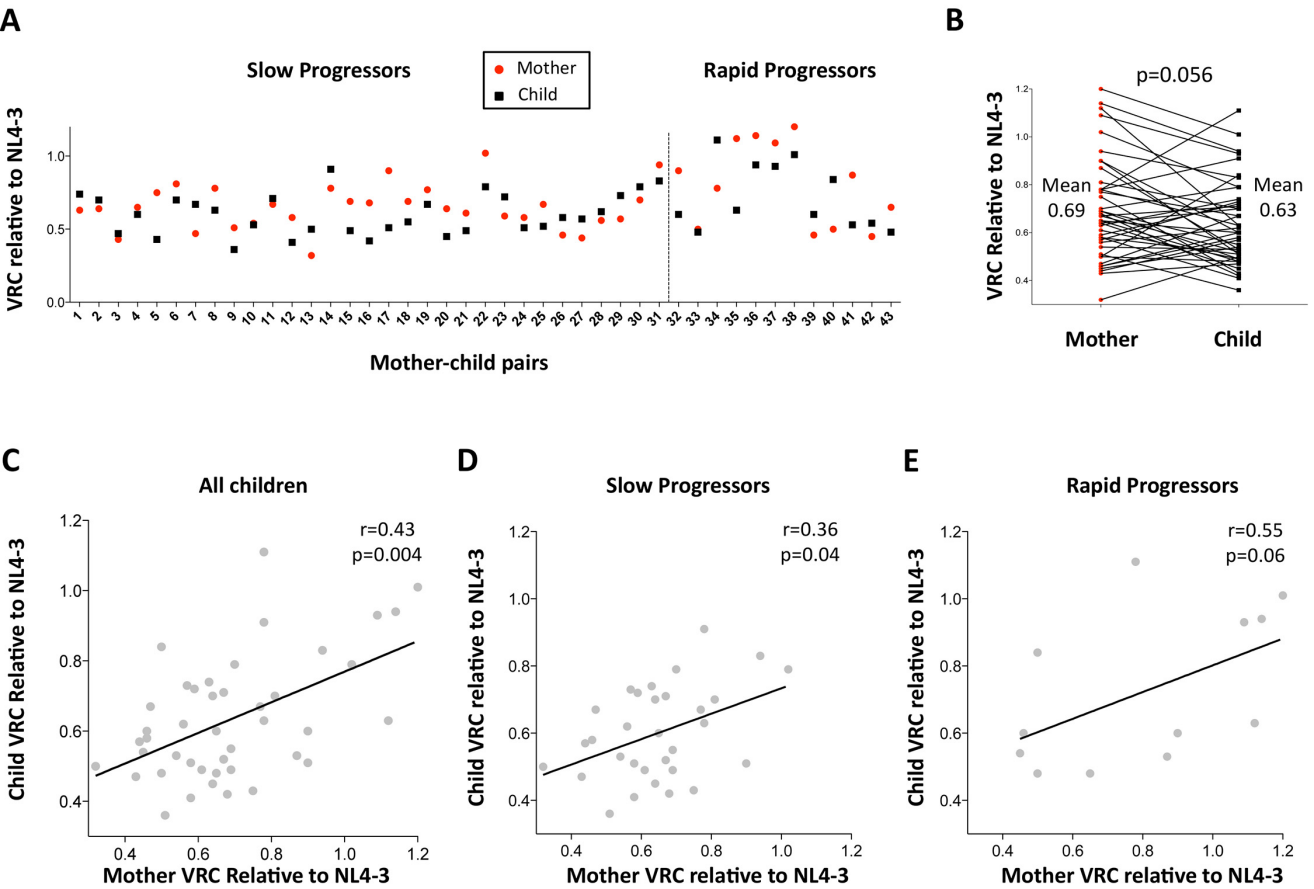
Relationship between maternal VRC and pediatric VRC. A. Maternal VRC (red circles) and child VRC (black squares) for each mother-child transmission pair. B. Comparison between VRC in mother and child: maternal mean VRC 0.69 versus VRC in the child 0.63; p = 0.056, paired t test. C. Correlation between VRC in mother and child for all children (n = 43, r = 0.43, p = 0.004). D. Correlation between VRC in mother and child for slow progressor children (n = 31, r = 0.36, p = 0.04). E. Correlation between VRC in mother and child for rapid progressor children (n = 12, r = 0.55, p = 0.06).

First, the authenticity of the mother-child transmission pairs was validated by phylogenetic analysis of all the viral sequences (S3 Fig). The VRC of chimeric viruses derived from the children was then compared with those of the respective mothers. Overall, somewhat surprisingly, the VRC in the mothers tends to be slightly higher than in the children (mean VRC 0.69 versus 0.62; p = 0.056, paired t test, Fig 2A and 2B). This result is discussed further below. However, considerable variation in this pattern was observed. As expected, overall, maternal and child VRCs were significantly correlated (r = 0.43, p = 0.004; Fig 2C), in spite of the fact that on average 7.5yrs had elapsed since transmission in the case of the slow progressors. In keeping with the fact VRC in the rapid progressors was determined from children and mothers a mean of only 14 months post transmission, the Spearman correlation coefficient between VRC in mothers and children was higher for the rapid progressors than for the slow progressors (r = 0.55 versus r = 0.36, respectively; Fig 2D and 2E).

### Differential impact of HLA Class I expression on VRC in adult and pediatric infection

**Fig 3:**
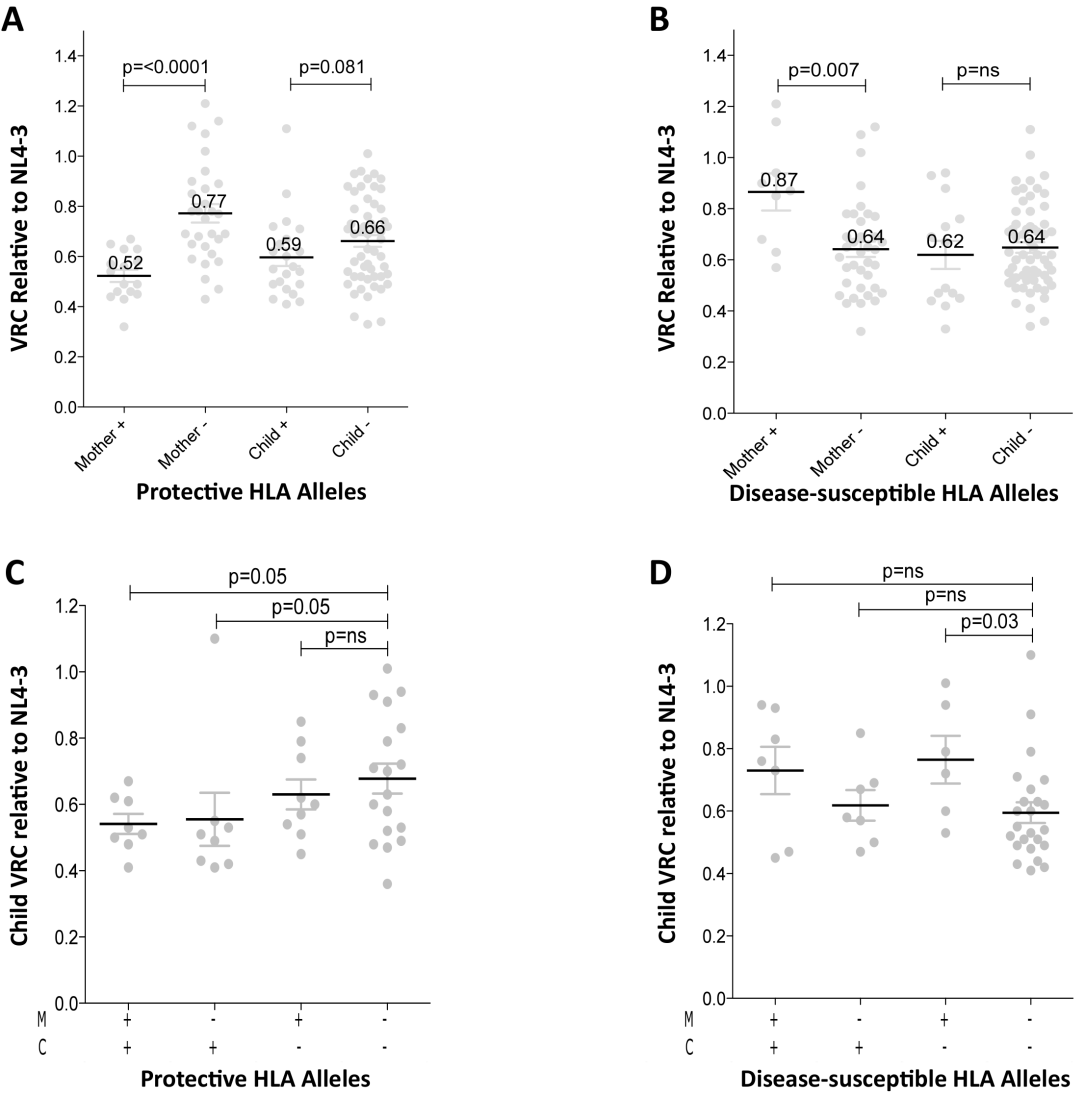
Relative contribution of HLA-B alleles in VRC in mothers and children. A. VRC in mothers expressing protective HLA alleles (n = 16) and not (n = 30); and in children expressing protective HLA alleles (n = 22) and not (n = 54). B. VRC in mothers expressing disease-susceptible HLA alleles (n = 9) and not (n = 37); and in children expressing disease-susceptible alleles (n = 14) and not (n = 62). C. VRC in the child according to expression of protective HLA alleles in mother or child (+/+ n = 8;-/+ n = 8; +/- n = 9;-/- n = 18). D. VRC in the child according to expression of disease-susceptible alleles in the mother and/or child (+/+ n = 7;-/+ n = 7; +/- n = 6;-/- n = 23).

In order to better understand the observed differences in VRC within mother-child pairs we next investigated the relative impact on VRC in mother and child of the protective HLA alleles HLA-B*57, HLA-B*58:01 and HLA-B*81:01, previously shown to significantly reduce VRC [12–20]. The VRC of chimeric viruses was substantially lower (VRC 0.52 vs 0.77) in the mothers who expressed any of these protective HLA alleles, compared with the mothers who did not (p<0.0001, Mann-Whitney test, Fig 3A). In contrast, there was a marginal impact on VRC of these protective HLA alleles in children (VRC 0.59 versus 0.66; p = 0.08). Similarly, in mothers who expressed one or more of the HLA alleles associated with more rapid disease progression, HLA-B*18:01, HLA-B*45:01, and HLA-B*58:02, VRCs were significantly higher than in those who did not express these alleles (VRC 0.87 vs 0.64; p = 0.007) whereas in children the VRC was not increased in those with disease-susceptible HLA alleles (VRC 0.62 vs 0.64, p = 0.41, Fig 3B). Thus the effects of the protective HLA alleles and disease-susceptible HLA alleles on VRC were striking among the adult subjects and minimal to absent in the children.

To further investigate the previous finding that slow progression is significantly associated with the mother or the child possessing one of the protective HLA-B alleles, but less so if the protective allele was shared by mother and child [30,33] we analyzed the VRC in children according to the presence or absence of protective alleles (HLA-B*57, HLA-B*58:01 or HLA-B*81:01) in mother and/or child. We observed here that, in the group where children and mothers both lacked protective alleles, the VRC was higher than the other three groups, significantly so where the mother and child share a protective allele and when the child alone carries the protective allele (p = 0.05) (Fig 3C). These data suggest that, although protective HLA alleles have a greater impact on VRC in adults than in children (Fig 3A), the VRC in children is more influenced by the protective alleles when they are expressed in the children themselves than when expressed in the mothers (Fig 3C). In contrast, the impact of the disease-susceptible alleles HLA-B*18:01/45:01/58:02 on the child’s VRC was greatest when the mother carried any of these (Fig 3D). Disease-susceptible alleles in the children therefore appeared to have little impact on VRC in the child.

### Strong correlation between Gag escape mutant number and VRC

**Fig 4:**
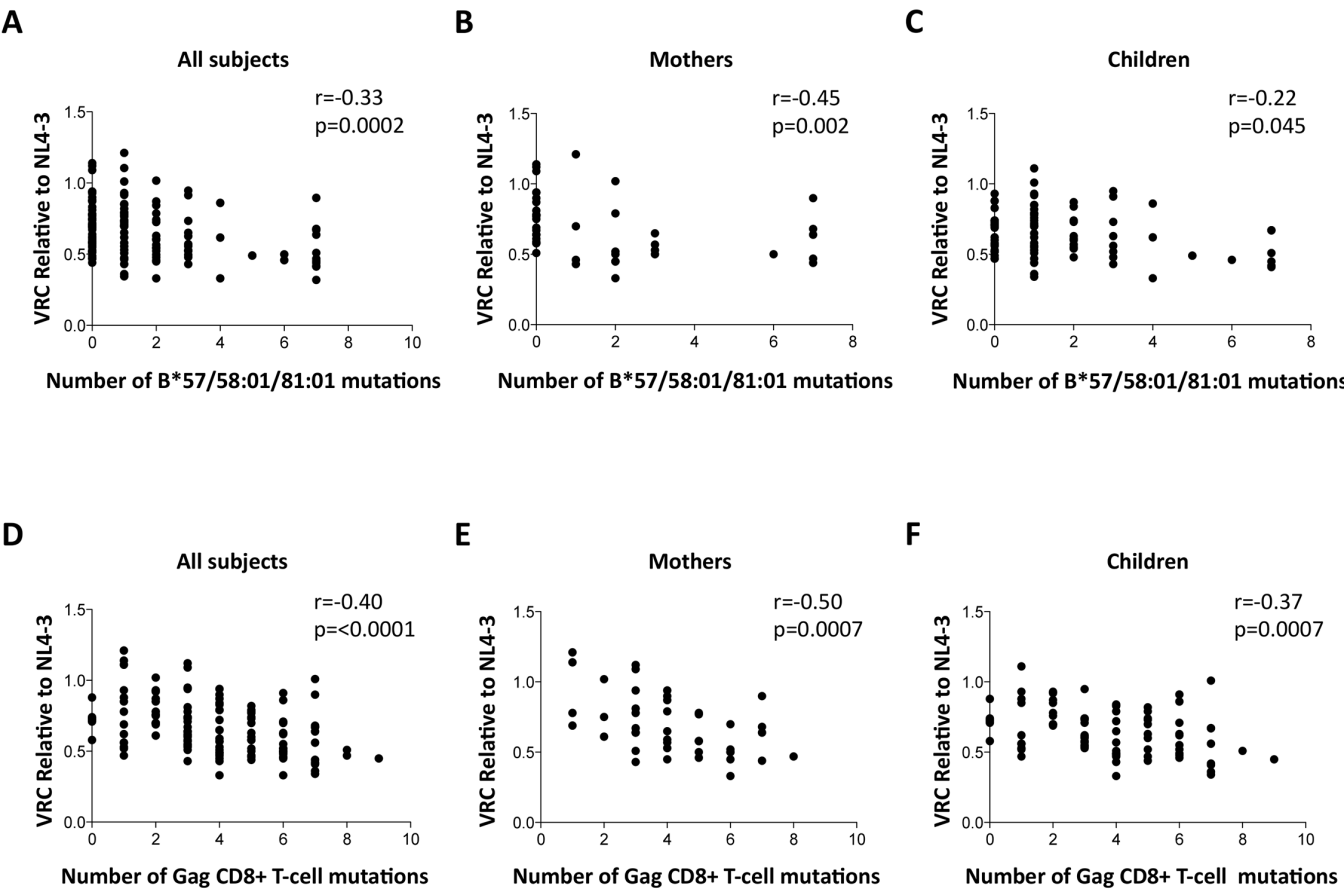
Impact of Gag mutations on VRC. A-C: HLA-B*57/58:01/81:01 mutations. (A146X, I147X, A163X, S165X, E177X, Q182X, T186S, T242X, I247X and T310X). A. All subjects; B. Mothers; C. Children. D-F: Gag CD8+ T-cell mutations (listed in S1 Table). DS. All subjects. E. Mothers. F. Children.

To determine whether the differences in VRC observed were indeed related to the occurrence of escape mutants in Gag selected by these protective HLA alleles as indicated by previous work^,^ [11–20,45,46] we sought a correlation between VRC and number of Gag escape mutants associated with these HLA alleles in each of the chimeric viruses studied, irrespective of the study subject. As expected, a highly significant correlation was observed between the number of Gag escape mutants associated with the protective HLA alleles and decreasing VRC (r = -0.33, p = 0.0002, Fig 4A). Breaking these data down into the mothers and children, the impact on VRC of these Gag mutants associated with the protective HLA molecules appeared greater in the mothers (r = -0.45, p = 0.002, Fig 4B) than the children (r = -0.22, p = 0.045, Fig 4C). However the impact of these Gag escape mutants on VRC in the slow progressor children was similar to that in the mothers (r = -0.37, p = 0.006); only in the rapid progressor children, where the number of Gag mutants associated with protective HLA alleles was low, was there no evidence of any impact of these HLA molecules on VRC (S4A and S4B Fig).

These analyses were repeated to assess the impact, not only of the Gag mutants associated with the protective HLA molecules HLA-B*57/58:01/81:01, but of all 20 Gag escape mutants within defined Gag CD8+ T-cell epitopes [11,15,45,46] (S1 Table). These include a number of mutants that it may be inferred reduce viral replicative capacity from evidence that these revert in the absence of the relevant HLA molecule [12,13,15] and reflect the impact of HLA molecules such as HLA-B*42:01, HLA-B*39:10 and HLA-B*14:01 that offer some degree of protection against disease progression, though not as much as provided by HLA-B*57/58:01/81:01 [2,7–8,11]. In contrast, the disease-susceptible HLA molecules make no contribution to the selection of Gag mutants. These analyses suggest that the additional Gag mutants also contribute to VRC, with overall a strong correlation between VRC and number of Gag CD8+ T-cell epitope mutants (r = -0.40, p<0.0001, Fig 4D); similar in mothers (r = -0.50, p = 0.0007, Fig 4E) as in children (r = -0.37, p = 0.0007, Fig 4F).

### Low Gag mutant number in adults with high VRC expressing disease-susceptible HLA

**Fig 5:**
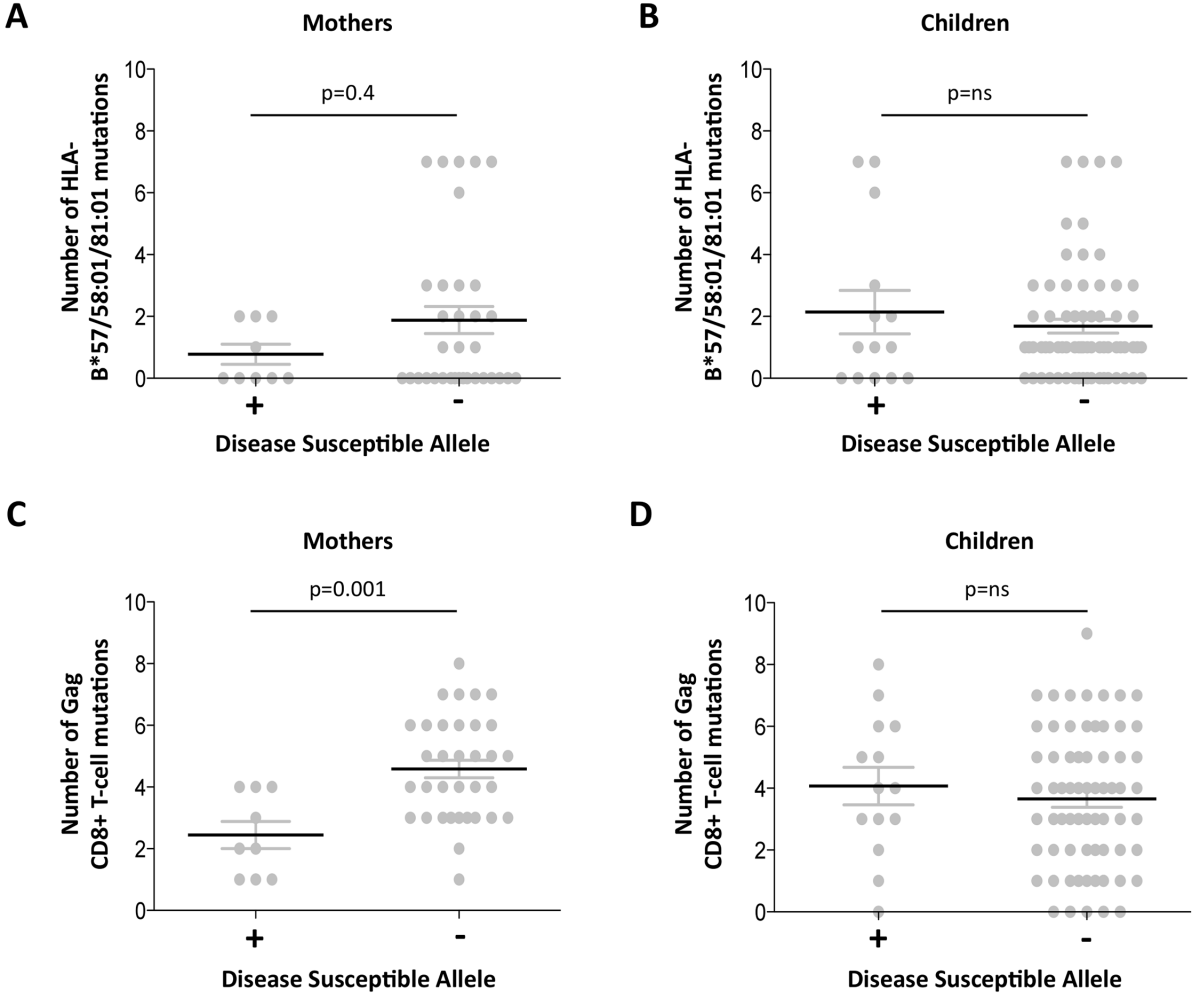
Gag mutant number in adults with high VRC expressing disease-susceptible HLA. A-B: HLA-B*57/58:01/81:01 mutations. A. Mothers. B; Children. C-D: Gag CD8+ T-cell mutations. C. Mothers. D. Children.

The observation of a clearcut increase in VRC in mothers expressing disease-susceptible alleles HLA-B*18:01/45:01/58:02 (shown in Fig 3B) prompts the question of what drives the VRC up in these subjects; or whether, alternatively, the VRC is driven down in those who do not express these alleles. Analysis of the number of Gag variants, using all 20 Gag escape mutants, in subjects who do and who do not express these disease-susceptible alleles, respectively, shows a substantially lower number of Gag escape mutants in the mothers expressing disease-susceptible alleles (mean 2.4 versus 4.6; p = 0.001, Student *t* test; Fig 5). This difference persisted even when subjects expressing the protective HLA alleles were omitted (p = 0.003,S5 Fig). These differences were also marked when the analysis was limited to the Gag mutants associated with the protective HLA alleles (mean 0.8 versus 1.9 in subjects who did and who did not express disease-susceptible alleles, respectively) although in this case this did not approach statistical significance (p = 0.4; Fig 5). In summary, these data are consistent with high VRC in adult subjects expressing disease-susceptible allele as the result principally of these HLA molecules failing to drive the selection of Gag CD8+ T-cell escape mutants. In contrast with the adults, in children the effects of these disease-susceptible alleles is apparently nil, with Gag escape mutants equally distributed between children who do and who do not express these HLA alleles.

### Discordant impact of HLA on progression in HIV-infected children and adults

**Fig 6:**
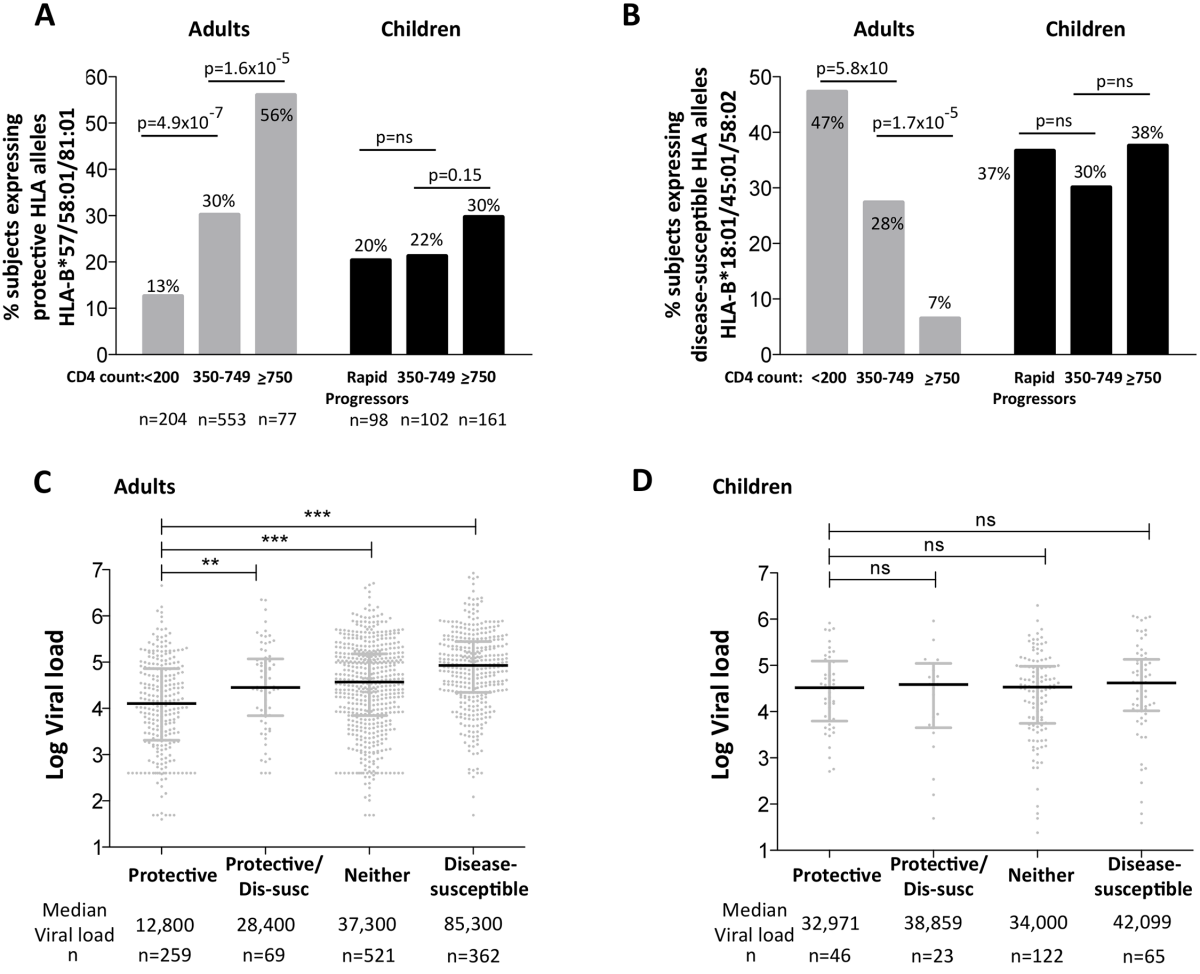
Impact of protective and disease-susceptible HLA alleles on disease progression in adults in children. A. The frequency of ART-naïve adults and children stratified according to CD4 count and disease progression (see Methods) expressing one or more of the protective HLA alleles HLA-B*57/58:01/81:01. Adult study numbers: absolute CD4<200/mm^3^, n = 204; CD4 350–749/mm^3^, n = 553; CD4≥750/mm^3^ n = 77. Children study numbers: Rapid Progressors, n = 98; CD4 350–749/mm^3^, n = 102; CD4≥750/mm^3^, n = 161. B. As in panel A, but the frequency of subjects expressing one or more of the disease-susceptible HLA alleles HLA-B*18:01/45:01/58:02. C. Viral load in ART-naïve adults (total, n = 1,211) according to the presence of protective HLA alleles and/or disease-susceptible alleles. ** represents p<0.001, *** represents p<0.0001. Not shown on the figure are significant differences between viral loads in the disease-susceptible group and each of the other groups (in each case p<0.0001). D. As in panel C, but in ART-naïve children (total n = 256, age ≥5yrs).

To compare the impact of protective and disease-susceptible HLA alleles on progression in adults and children we first analyzed the expression of these alleles in chronically infected, ART-naïve South African adults, analyzed according to absolute CD4 count at enrollment (Fig 6A and 6B). As expected, protective HLA alleles were highly enriched in adults with high CD4 counts and disease-susceptible alleles highly enriched in adults with low CD4 counts. In HIV-uninfected children during the first five years of life, absolute CD4 counts change dramatically according to age, declining from a median of 2500–3000/mm^3^ during the first year to a stable median of approximately 750–1000 cells/mm^3^ from age 5yrs through adulthood [41]. Comparing ART-naïve, HIV-infected children aged >5yrs with CD4 counts of ≥750 cells/mm^3^ with those with CD4 counts of 350–749 cells/mm^3^, we observed no significant difference in the frequency of protective or disease-susceptible HLA alleles in these groups. Similarly, in a rapid progressor group of HIV-infected children (median age at ART initiation 6m, median absolute CD4 count 584/mm^3^, median CD4% 13%, and median viral load 1.1m), the frequency of protective and disease-susceptible alleles also did not differ significantly from the ‘slow progressor’ groups of infected children. Thus, the impact of the protective and disease-susceptible HLA alleles on CD4 count is marked in adult infection, but is barely discernible in pediatric infection. Similarly, analysis of the impact of the protective and disease-susceptible HLA alleles on viral loads in ART-naïve adults and children (Fig 6C and 6D) showed significant effects among the adults (a median 0.8 log HIV copies/ml difference between adults with protective alleles versus those with disease-susceptible alleles), and insignificant effects among the slow progressor children. Although, as described above, viral load is less helpful as a proxy for disease progression in pediatric versus adult infection, it is clear that in terms of the biological markers of disease progression, absolute CD4 count and viral load, the HLA alleles that have the strongest effects on outcome in adult infection have minimal impact in pediatric infection.

### Lack of selection pressure mediated by protective HLA in rapid progressor children

In order to help explain the diminished impact of particular HLA allele expression in infected children, we sought to test the hypothesis that the CD8+ T-cell responses restricted by protective HLA molecules such as HLA-B*57/58:01/81:01 generally have less antiviral efficacy in children compared to adults. The selection of viral escape mutants to evade the dominant Gag-specific CD8+ T-cell responses restricted by these alleles is evidence of immune selection pressure, and is observed in 60–80% of unselected, chronically adults expressing these alleles [11–13,46]. Hypothesizing that anti-HIV CD8+ T-cell efficacy would be significantly lower, and therefore that the frequency of these escape mutants would also be substantially lower, in rapidly progressing infants expressing any of these ‘protective’ HLA molecules, we studied a historical cohort of 20 ART-naïve, HIV-infected infants born in Durban, South Africa, between 2002–2005 [42], who were followed from birth. Antenatal, third trimester samples in each case were obtained from the transmitting mothers, providing close approximations to the transmitted virus. A current day cohort of this type would not be possible to study because since 2008 WHO guidelines have recommended ART initiation in all HIV-infected infants, irrespective of CD4% [52,53]. In this historical cohort, the CD4 criteria for ART initiation in infants followed the prevailing WHO guidelines, namely a confirmed CD4% of <20% [54].

**Fig 7:**
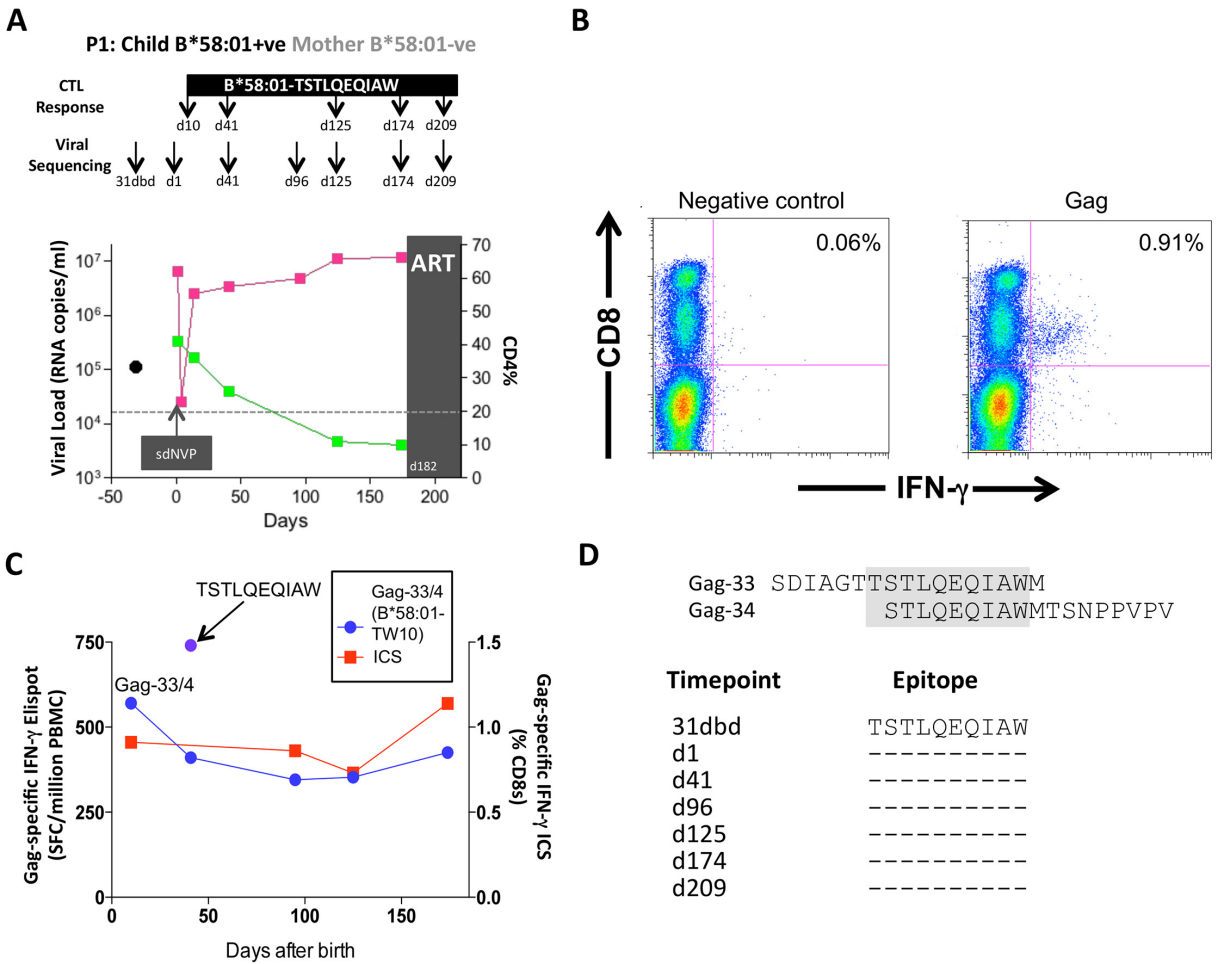
Gag-specific CD8+ T-cell responses and autologous viral sequences in a rapid progressing child, P1. A. Viral load and CD4% changes, CTL assays and viral sequencing timepoints. Closed red squares: viral load. Closed green squares: CD4%. Horizontal dotted lie: threshold for ART initiation (confirmed CD4<20%). Black closed circle: timepoint of maternal viral sequencing. Dbd: days before delivery. sdNVP: single dose nevirapine, given during labour and on the first day of life to the infant. D182: age of child at ART initiation. B. Gag-specific intracellular interferon-gamma staining assay undertaken when P1 was 10 days of age. C. Interferon-gamma elispot responses to Gag-33 and Gag-34 (two of 66 overlapping 18mers spanning the Gag protein), and on day 41 to the HLA-B*58:01-retricted Gag epitope TSTLQEQIAW contained within overlapping peptides Gag-33/4; and Gag-specific IFN-γ ICS data, showing responses to 66 pooled overlapping Gag peptides. D. Maternal viral sequence 31 days before delivery and autologous viral sequence in P1 showing lack of escape in all time points analyzed from day 1 to day 207, 25 days after ART initiation.

Of these 20 children, 17 met the CD4 criteria for ART initiation within the first year of life (confirmed CD4%<20%), thereby also meeting the criteria in this current study for rapid progression (CD4%<25% within the first 24m of life). Five of these 17 rapid progressor children carried one of the protective HLA molecules HLA-B*57/58:01/81:01. For convenience, these children are referred to here as P1-P5. Children P1-P2 each expressed HLA-B*58:01 and the mothers did not express HLA-B*58:01; as expected, therefore, in each case, the transmitted virus encoded no mutations within the dominant HLA-B*58:01-restricted Gag epitope, TSTLQEQIAW (‘TW10’; Gag 240–249). In P1, a strong CD8+ T-cell response was detectable towards 66 pooled overlapping 18mer Gag peptides when first assayed on day 10 of life (Fig 7A and 7B); individual testing of the overlapping Gag peptides demonstrated that the single Gag-specific response was towards the two 18mer peptides (Gag-33/Gag-34) containing the TW10 epitope in the 10 amino acid overlap (Fig 7C and 7D). Other than the short-lived drop in viremia [55] resulting from single dose Nevirapine that each child received on the day of birth, and the mother received during labour, as per prevailing prevention of mother-to-child transmission guidelines at the time [56], viral load in this child increased from 6.5m copies/ml on day 1 of life to >10m copies/ml over the first 6 months of life, coincident with maintenance of the TW10/Gag-specific response at the same level of 0.73–1.14% CD8+ T-cells. However, throughout this time there was no evidence of selection of TW10 or other Gag escape mutants (Fig 7D).

**Fig 8:**
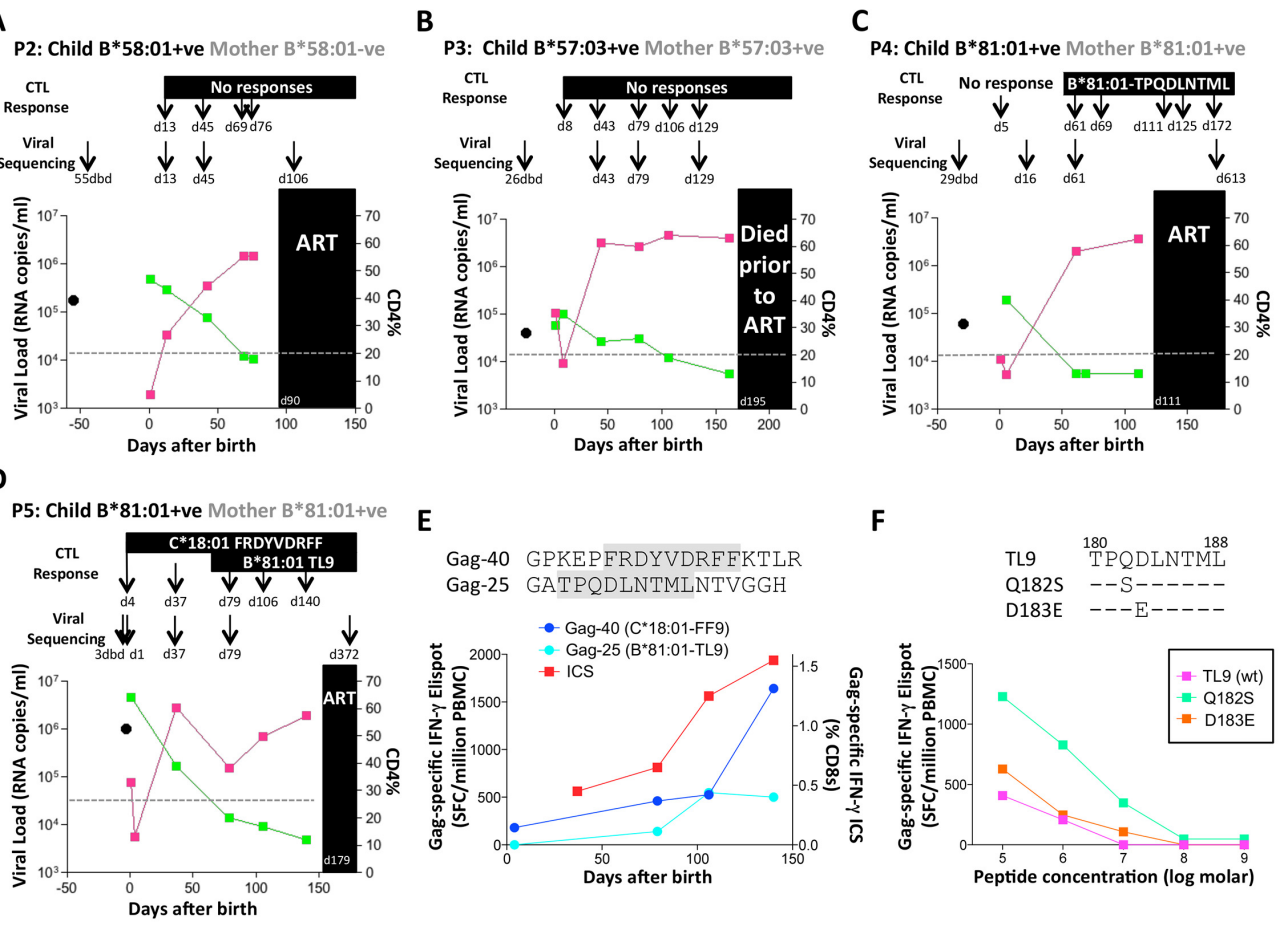
Rapid progression and lack of CTL escape in 4 additional rapid progressor expressing protective HLA-B*57/58:01/81:01. A-D. Clinical data for subjects P2-P5 (as for P1 in Fig 7A). E. In child P5, elispot IFN-γ responses to 17–18mer Gag peptides Gag-40 (from day 4 of life) and Gag-25 (from day 79 of life) and Gag ICS responses to the pool of 66 Gag overlapping peptides. F. In child P5, recognition in IFN-γ elispot assays of autologous TL9 variants TPSDLNTML (Q182S) and TPSELNTML (D183E), compared to recognition of wildtype TL9. PBMC were tested at day 79 and day 141, with the pattern; data are shown from PBMC at day 141.

No HLA-B*58:01-TW10 or Gag escape mutants were selected in P2, although in this case no CD8+ T-cell responses were detected to any of the same set of Gag peptides (Fig 8A and Table 1). In the HLA-B*57:03-positive child, P3, although the mother also expressed HLA-B*57:03, the transmitted virus did not carry escape mutants in the HLA-B*57:03-restricted Gag epitope KAFSPEVIPMF (‘KF11’, Gag 162–172). In this child, no KF11 CD8+ T-cell responses were detected and no escape mutants selected (Fig 8B and Table 1). In the HLA-B*81:01-positive child, P4, the transmitted virus did not carry escape mutants in the HLA-B*81:01-restricted Gag epitope TPQDLNTML (‘TL9’, Gag 180–188). In this case, strong TL9-specific CD8+ T-cell responses were consistently detectable at >1000 SFC/million PBMC (1162 and 1073 SFC/million PBMC on day 61 and day 69, respectively), but progression to CD4%<20% with rising viremia occurred without selection of TL9 escape mutants (Fig 8C and Table 1).

**Table 1:** Autologous virus sequences in children P2-P5 and their mothers encoding the relevant Gag-specific CD8+ T-cell epitopes restricted by HLA-B*57/58:01/81:01 and HLA-C*18:01.

Progressor Pair		Timepoint^a^		Epitope	and HLA	Restriction	
			B*57:03	B*57:03	B*81:01	B*57/58:01	C*18:01
			AISPRTLNAW	KAFSPEVIPMF	TPQDLNTML	TSTLQEQIAW	FFRDYVDRF
**P2 Mother**	B*58:01-ve	55dbd	N/A	N/A	N/A	----------	N/A
**P2 Child**	B*58:01+ve	d13	N/A	N/A	N/A	----------	N/A
		d45	N/A	N/A	N/A	----------	N/A
		d106	N/A	N/A	N/A	----------	N/A
**P3 Mother**	B*57:03+ve	26dbd	PL--------	-----------	N/A	--N-------	N/A
**P3 Child**	B*57:03+ve	d43	PL--------	-----------	N/A	--N-------	N/A
		d79	PL--------	-----------	N/A	--N-------	N/A
		d123	PL--------	-----------	N/A	--N-------	N/A
**P4 Mother**	B*81:01+ve	29dbd	N/A	N/A	---------	N/A	N/A
**P4 Child**	B*81:01+ve	d18	N/A	N/A	---------	N/A	N/A
		d61	N/A	N/A	---------	N/A	N/A
		d613	N/A	N/A	---------	N/A	N/A
**P5 Mother**	B*81:01+ve	3dbd	N/A	N/A	--SX------	N/A	---------
**P5 Child**	B*81:01+ve	d1	N/A	N/A	---E------	N/A	---------
		d37	N/A	N/A	--S-------	N/A	---------
		d79	N/A	N/A	--S-------	N/A	---------
		d372	N/A	N/A	--S-------	N/A	---------

^a^ timepoint: dbd = days before delivery of child; X: mixed bases; N/A: not applicable - relevant HLA allele not expressed

The fifth case, P5, was more complex to evaluate, because both mother and child expressed HLA-B*81:01, and the virus sequenced from the mother 3 days prior to delivery encoded a mixture of TL9 variants (Fig 8D, 8E and 8F and Table 1). The dominant variant in the child switched from D183E on day 1 of life to Q182S by day 37. A response to the Gag 18mer (Gag-25) containing TL9 was not detectable until day 79 (Fig 8D, 8E and 8F), and assays at this time point and at day 141 just prior to ART initiation showed the same pattern, namely, no evidence that Q182S was an escape mutant, but that Q182S was better recognized than both the D183E variant and the wildtype TL9 peptide (Fig 8F). The evidence that TL9 escape occurred in child P5 is therefore equivocal, at best. It is clear however that the dominant Gag response in this child, that was detectable from day 4 in life, was to the HLA-*18:01-restricted epitope FRDYVDRFF (‘FF9’, Gag 293–301) [57]. This response, although like the TL9-Q182S response, present at >1000 SFC/million PBMC in elispot assays, also did not drive selection of escape mutants over the 6 months of disease progression towards ART initiation.

In summary, these 5 cases of HIV-infants followed from birth progressed rapidly to CD4% of <20%, in the setting of persistent viremia of >10^6^−10^7^ copies/ml, in spite of expression of the protective HLA alleles HLA-B*57/58:01/81:01, and did so in a manner notable by the absence of the selection of escape mutants. In some cases these children did not generate detectable CD8+ T-cell responses, but in others these responses were maintained at relatively high frequencies.

## Discussion

These studies of HIV-infected children and mothers show, first, a significant correlation between the replicative capacity of viruses in infected children and their rate of progression. Second, despite the fact that, on average, 7.5yrs had elapsed since transmission, there remained a highly significant correlation between the VRC in mothers and children within transmission pairs. Unexpectedly, the VRC was typically lower in the child than in the mother within each pair. Third, the impact of protective HLA alleles HLA-B*57/58:01/81:01 and disease-susceptible HLA alleles HLA-B*18:01/45:01/58:02 on VRC was marked among the adults but non-significant or absent, respectively, among the children. However, the impact of maternal alleles on the child’s VRC was greatest when the mother carried any of the disease-susceptible alleles HLA-B*18:01/45:01/58:02. The impact of protective and disease-susceptible alleles on VRC was strongly linked to the number of Gag CD8+ T-cell escape mutations within the virus. Finally, the impact of protective and disease-susceptible HLA alleles on disease progression, using CD4 count or viral load as a marker, was strikingly discordant between children and adults, with minimal HLA impact discernible among children. Longitudinal analysis from birth of 5 HIV-infected children who progressed rapidly in spite of expressing protective HLA alleles showed that even when high frequency responses restricted by the relevant protective HLA alleles were generated, no evidence for any impact on viral replication was evident. Together, therefore, these data are consistent with a substantially diminished impact of HLA on disease outcome in pediatric infection compared to the marked effect observed in adults, although the influence of maternal HLA, principally via VRC of the transmitted virus to the child, does also affect disease progression in children.

The central part HLA plays in immune control of adult HIV infection has long been evident, and the more recent GWAS studies have served to highlight the exclusivity of HLA polymorphism among human genetic variation in shaping HIV disease outcome [2–4]. Numerous mechanisms by which HLA may mediate these effects have been proposed [5], prominent among which is the hypothesis that protective HLA alleles such as HLA-B*27/57/58:01/81:01 reduce viral replicative capacity (VRC) through driving the selection of escape mutants in conserved regions of the proteome such as Gag [12–20]. The high throughput assay developed to measure VRC using Gag-Protease recombinant viruses [47] is therefore well-suited to examine the impact of HLA on VRC through the selection of escape mutants largely arising in Gag.

Although in previous studies the relationship between VRC as measured by this assay and the viral escape mutants selected in response to Gag CD8+ T-cell activity has been somewhat inconsistent [18,45,49,51], here the correlation between the number of Gag escape mutants and VRC has been very clear (in adults, r = -0.50, p<0.001 when including all Gag CD8 T-cell escape mutants; and r = -0.45, p = 0.002 when including only the HLA-B*57/58:01/81:01-associated Gag escape mutants). Taking into account that the 20 Gag CD8+ T-cell escape mutants are only those that represent the strongest HLA footprints on the virus, and therefore meet the statistical criteria (usually q<0.05 or q<0.2) [11,15,45] to be included, the likelihood is that the selection of Gag escape mutants by CD8+ T-cell responses has a substantially greater impact on VRC even than these data would indicate. Furthermore, the correlations between VRC and number of Gag escape mutants would diminish and ultimately disappear if in the majority of cases compensatory mutants were successfully selected by the virus to nullify any adverse impact on VRC. On the basis of these data, the ability of compensatory mutants to restore VRC appears limited. If so, this would be consistent with recent data suggesting that immune pressure mediated by protective alleles such as HLA-B*57/58:01 is contributing to a decline in HIV virulence over time [45].

The present studies address the role of HLA and of VRC in adult and pediatric HIV infection. It is known that VRC affects outcome in adult infection, as previously shown [37,40]. Consistent with the only other study that has examined VRC in pediatric HIV infection, in a small study of 8 infected children [35], here we show that VRC is also related to pediatric disease outcome (Fig 1), being on average 15% lower in slow progressors compared to rapid progressors, and correlating with CD4 count at 5yrs and 7.5yrs age, despite the VRC assays in the slow progressor children being determined on average 7.5yrs after transmission.

Unexpectedly, the VRC in mothers of the 43 mother-child pairs studied tended to be somewhat higher than in the children (mean VRC 0.69 vs 0.63; p = 0.056). In some cases, this result might be explained by the accumulation of costly escape mutants in the child’s virus post-transmission; however, the same result was obtained from a separate study of VRC in 39 mother-child pairs in which the VRCs were determined from samples taken from mother and child within 6 weeks of birth [58]. In that study, the VRC of the infected neonates was on average 20% lower than that in the mothers (median VRC 0.62 vs 0.77; p<0.0001). The fact that both these results are consistent in indicating that the virus in the child has lower VRC than in the mother suggests either that intense selection pressure is imposed on HIV in the initial weeks on perinatal infection, which seems unlikely [59]; or that lower VRC viruses are preferentially transmitted. If the latter, this would be the exact opposite of adult sexual transmission, in which a transmission bias exists against viruses with low VRC [60]. This discrepancy remains unexplained, and requires further investigation, but may indicate a distinct bottleneck in operation in perinatal mother-to-child-transmission compared to adult horizontal transmission.

The discordant relationship between HLA and VRC in infected adults and children (Fig 3) highlights the substantial impact of HLA on VRC in adults but not in children. Although protective alleles in the child appear to effect a modest reduction in VRC (Fig 3A and 3C), the protective alleles in the mother do not appear to have significant influence on the VRC in the child, consistent with one previous study [34] and in contrast to another [33]. However the existence of disease-susceptible alleles in the mother had a significant impact on VRC not only in the mother but also in the child. The mechanism by which these disease-susceptible HLA alleles might operate is suggested by the data showing a significantly lower number of Gag CD8+ T-cell escape mutants in mothers expressing these alleles than those not. This supports the hypothesis that disease susceptible alleles are ineffective against HIV principally because they fail to contribute to Gag-specific immune pressure on the virus, rather than because they actively mediate immune responses that prevent effective antiviral control [61,62].

Comparison of the relationship between HLA and disease outcome in infected adults and children (Fig 6) serves to contrast the striking influence of HLA in adults with the relative lack of impact in children. Protective and disease-susceptible HLA alleles had no statistically significant effect on disease progression in infected children, on CD4 counts or on viral loads. Although previous studies have anecdotally described slow disease progression in HIV-infected children expressing protective HLA alleles such as HLA-B*27 or HLA-B*57 [30,31,34], less prominently the same result has also been observed in children expressing no protective alleles [32,36]. It seems clear that, in a minority of slow progressor children expressing the classical protective HLA alleles, HLA-B*57/58:01/81:01, the same Gag-specific CD8+ T-cell responses are generated as have been well-described in adults, driving the same immune selection pressure, and in some cases these appear to contribute to slow progression in these children. There is evidence for this in the published literature [30,31,34], and also in the current study, in which we observed a modest impact on VRC in children expressing HLA-B*57/58:01/81:01 (Fig 3A and 3C), a moderate increase in children expressing these alleles in the slow progressors with the highest CD4 counts (Fig 6A), and examples of HLA-B*57/58:01/81:01 CD8+ T-cell pressure driving the selection of Gag escape mutants that reduce VRC (S2 Table), just as in adults. However, it is equally clear from these studies that the impact of these protective and also of the disease-susceptible HLA alleles is substantially less in pediatric than adult HIV infection.

Potential reasons for diminished HLA effect in children are suggested by the data presented showing rapid progression in children in spite of expressing the protective HLA-B*57/58:01/81:01 alleles and, at least in some cases, in spite of the classical Gag-specific CD8+ T-cell responses being detectable at highly respectable levels. The precise reasons why these CD8+ T-cell responses appear to have been ineffective in these children would be of interest to define further. However, a number of more general factors have been identified that mitigate against effective CD8+ T-cell responses being generated in infancy, foremost amongst these being the consequences of immune ontogeny [59], and the bias of CD4 T cells in infancy to make Th2 type responses. This in turn may reflect the inability of innate immune cells in the infant to produce IL-12, in contrast with high production of the immunomodulatory cytokine IL-10 [59].

It is important also to consider the limitations of this study. One of these is the fact that we assessed VRC in the rapid progressor children at mean age 14 months and in the slow progressor children at mean age 7.5 years. Ideally we would have measured VRC in the slow progressor children at the same age as in the rapid progressors. However this would have necessitated follow up of a cohort of HIV-infected children from birth, and most of these children were not identified until >5yrs of age. However, a small longitudinal study was previously undertaken by our group, of VRC in 8 HIV-infected children followed from birth, comprising 3 ‘progressors’ and 5 ‘slow progressors^’^ [35]. The conclusions of that longitudinal study, that VRC is higher in progressors than in slow progressors, and that the lower VRC observed in slower progressors is lower from early in the course of infection, are consistent with those of the current study. However, it would be useful to undertake longer follow up of VRC over the course of infection to determine whether any predictable pattern of VRC changes over time exists. On the one hand it might be expected that escape mutants accumulating over the course of infection might decrease VRC, whilst on the other hand the accumulation of compensatory mutants might increase VRC over time.

With regard to the other potential limitations of this study, it is clear that viral replicative capacity is not solely dependent upon mutations within *gag* and *pro* but also in genes elsewhere in the genome. Furthermore, it is important to note that a range of factors, in addition to CD8+ T-cells, operate to impose selection pressure on the virus, including neutralizing antibodies and influences affecting viral tropism. However, the justification for focusing on the Gag-Pro region as we have done here is as follows. First, there is strong evidence that Gag-specific CD8+ T-cell epitopes play a central role in immune control of HIV, and that the fitness cost of Gag escape mutants contributes to the impact of protective HLA molecules such as HLA-B*57 [5,12–20,47]. Although studies of the fitness cost of CD8+ T-cell escape mutations outside of Gag-Pro are less extensive than within Gag, the available evidence suggests that escape mutants in proteins such as Env are less likely to carry a fitness cost [24–26].

Second, in several studies, the biological relevance of the Gag-Pro assay has been demonstrated by the correlations with markers of disease progression (namely, absolute CD4 count and viral load) [18,45,47–51]. Thus, in order to examine the impact of specific HLA-associated sites within Gag, the optimal way to do that would using an isogenic backbone to generate Gag-Pro recombinant chimeric viruses as we have done here.

The third point in favor of this assay is that, in practical terms, generating a full-length replication competent clone from each subject isolate is a labor-intensive and costly process. This illustrates why, when we previously adopted that approach, in the only report to date to measure directly VRC in infected children, the study was limited to n = 8 [35]. In contrast, here we have determined VRC in more than 15 times that number of subjects. Finally, at the end of the process of isolating full-length viruses, unless many hundreds of virus clones are assayed, there are too may mutations across the whole viral genome to determine which has contributed to differences in replication capacity. A practical approach therefore to address the impact of HLA on VRC is the one adopted here.

Together these data suggest that VRC, and the impact that HLA has on VRC, plays an important role in disease progression in adult and pediatric infection. However, these data also would indicate that VRC is by no means the only factor that influences rate of disease progression in pediatric infection. Furthermore, in infected children, the effect of HLA on outcome that is independent of VRC appears to be limited. The difference in HLA impact between adults and children may reflect the fact that distinct mechanisms may operate to limit disease. In adults, the route to immune control and consequent disease limitation appears to lie, at least in part, in an effective CD8+ T-cell response, such as that restricted by protective HLA alleles, that can bring down viral load to low levels. In pediatric infection, non-pathogenicity is weakly related to HLA and viral load [63]. In contrast to adult infection, persistently high viral loads in pediatric slow progressors are observed in association with low levels of immune activation [63], with similarities to the natural hosts of SIV infection [64]. Pediatric slow progressors thus now provide the opportunity to address in humans a central, unanswered question in relation to HIV pathogenesis, namely, the mechanisms underlying low immune activation in the face of persistently high viremia.

## Supporting Information

S1 Fig Phylogenetic tree of Gag sequences derived from 33 patient plasma samples and Gag-Pro chimeric viruses. A. A maximum-likelihood tree was constructed using PHYm131 (http://www.hiv.lanl.gov). “C” denotes a child isolate and “M” donates a maternal isolate. Each mother-child pair carries the same number, for example K140C and K140M represent the K140 mother-child pair. The scale represents substitutions per site. V represents chimeric virus sequence, P represents plasma virus sequence. B. The deduced Gag amino acid sequences determined from plasma virus and from the Gag-Pro-chimeric virus were compared for each of the 33 subjects. The percent amino acid identity was determined for each subject after exclusion of codons including mixed bases, as previously described [18,45,47,48].
(TIF)

S2 Fig Relationship between viral load and VRC and absolute CD4 count in pediatric study subjects. A. Viral load versus absolute CD4 count in ART-naïve children of 5 yrs age. B. Viral load versus absolute CD4 count in ART-naïve children of 7.5 yrs age. C. Viral load versus VRC using viral loads at 5yrs age in pediatric study subjects. D. Viral load versus VRC using viral loads at 7.5yrs age in pediatric study subjects.
(TIF)

S3 Fig Phylogenetic tree of Gag sequences derived from patient plasma samples. A maximum-likelihood tree was constructed using PHYm131 (http://www.hiv.lanl.gov). “C” denotes a child isolate and “M” donates a maternal isolate. Each mother-child pair carries the same number, for example K140C and K140M represent the K140 mother-child pair. The scale represents substitutions per site.
(TIF)

S4 Fig VRC compared with number of Gag-specific CD8+ T-cell escape mutations in rapid and slow progressor children. A-B: HLA-B*57/58:01/81:01-associated Gag mutants. A. Slow progressors. B. Rapid progressors. C-D. Gag-specific CD8+ T-cell mutations within defined epitopes (listed in S1 Table). A. Slow progressors B. Rapid progressors.
(TIF)

S5 Fig Impact of Gag CD8+ T-cell mutations (listed in S1 Table) in adults expressing disease susceptible HLA. (TIF)

S1 Table Gag-specific CD8+ T-cell mutations within defined epitopes (refs 11, 15, 45, 46). (TIF)

S2 Table Representative autologous mother (M) and child (C) Gag sequences within epitopes restricted by protective HLA-B alleles B*57/B*58:01/81:01 to demonstrate occurrence of escape within selected slow progressor children. Mutations shown; A146X and I147X within the HLA-B*57 restricted epitope ISPRTLNAW (ISW9, Gag 147–155); A163X and S165X within the HLA-B*57-restricted epitope KAFSPEVIPMF (KF11, Gag 162–172); Q182X and T186S within the HLA-B*81:01-restricted epitope TPQDLNTML (TL9, Gag 180–188); and T242X and I247X within the HLA-B*57/58:01-restricted epitope TSTLQEQIAW (TW10, Gag 240–249).
(TIF)
