# Supplementary figures and images for: Discordant Impact of HLA on Viral Replicative Capacity and Disease Progression in Pediatric and Adult HIV Infection
Source: PLoS Pathog. 2015 Jun 15;11(6):e1004954. doi: 10.1371/journal.ppat.1004954 (PMC4468173; doi:10.1371/journal.ppat.1004954)

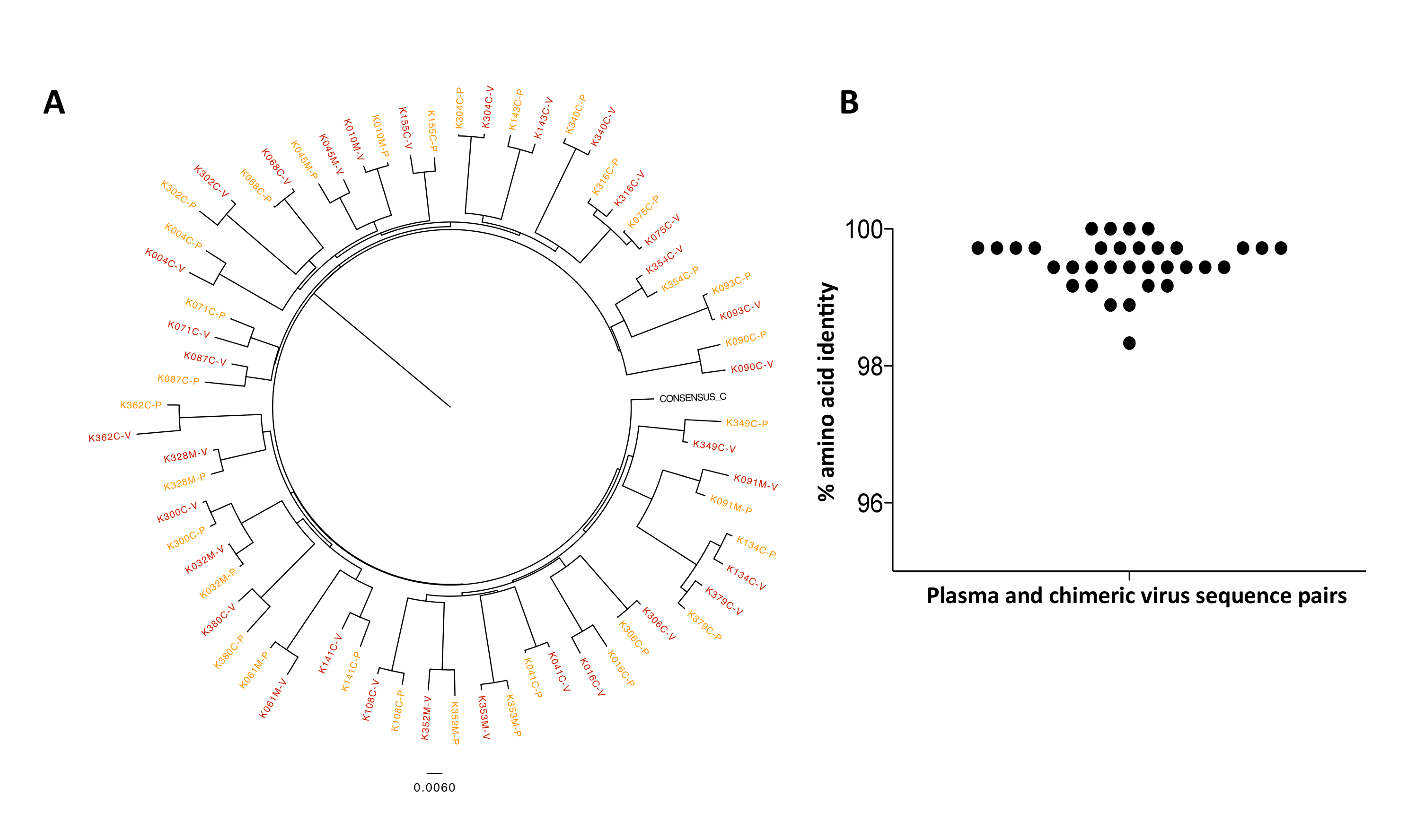

Supplement: S1 Fig — A. A maximum-likelihood tree was constructed using PHYm131 (http://www.hiv.lanl.gov). “C” denotes a child isolate and “M” donates a maternal isolate. Each mother-child pair carries the same number, for example K140C and K140M represent the K140 mother-child pair. The scale represents substitutions per site. V represents chimeric virus sequence, P represents plasma virus sequence. B. The deduced Gag amino acid sequences determined from plasma virus and from the Gag-Pro-chimeric virus were compared for each of the 33 subjects. The percent amino acid identity was determined for each subject after exclusion of codons including mixed bases, as previously described [18,45,47,48]. (TIF) [file ppat.1004954.s001.tif]

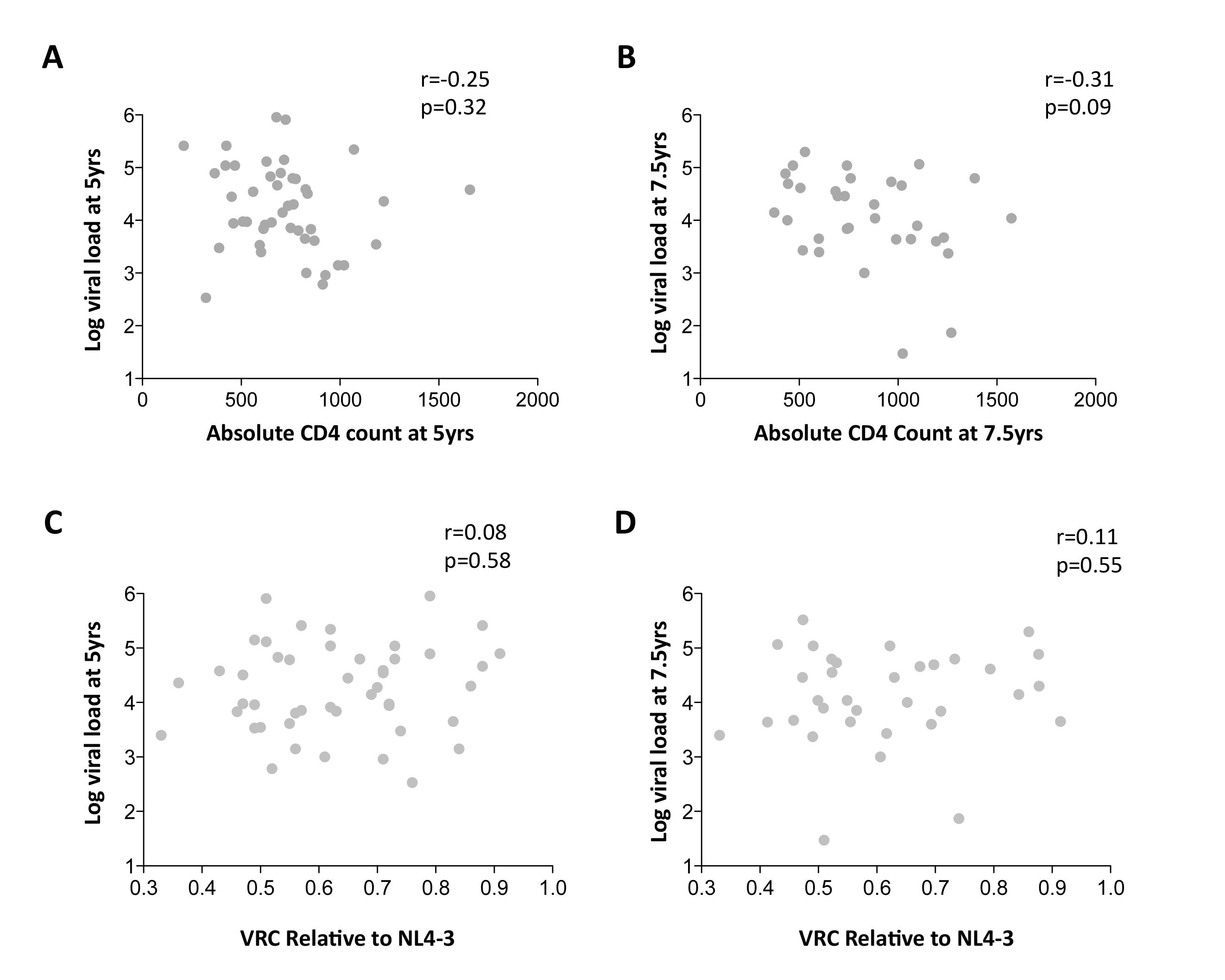

Supplement: S2 Fig — A. Viral load versus absolute CD4 count in ART-naïve children of 5 yrs age. B. Viral load versus absolute CD4 count in ART-naïve children of 7.5 yrs age. C. Viral load versus VRC using viral loads at 5yrs age in pediatric study subjects. D. Viral load versus VRC using viral loads at 7.5yrs age in pediatric study subjects. (TIF) [file ppat.1004954.s002.tif]

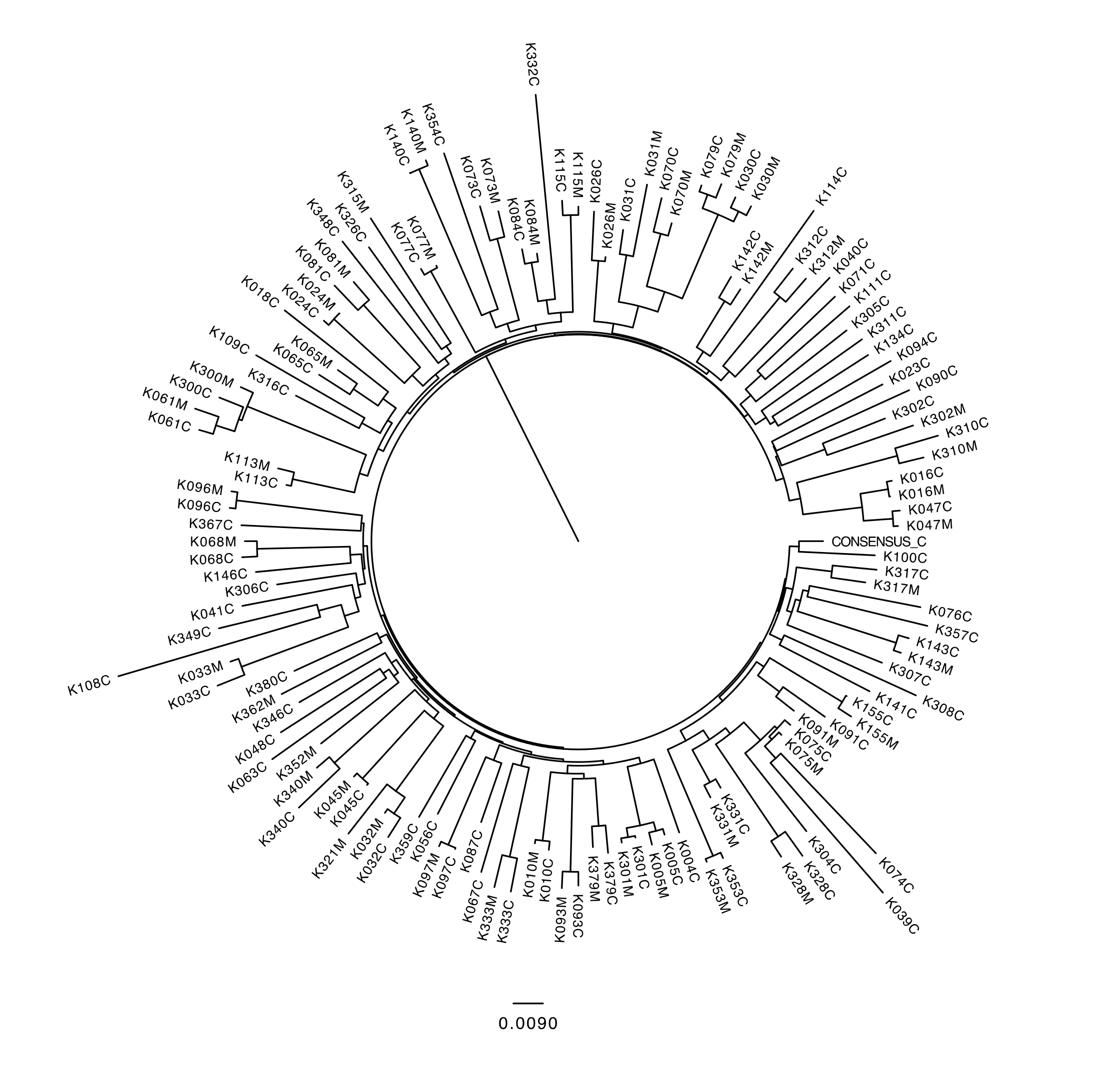

Supplement: S3 Fig — A maximum-likelihood tree was constructed using PHYm131 (http://www.hiv.lanl.gov). “C” denotes a child isolate and “M” donates a maternal isolate. Each mother-child pair carries the same number, for example K140C and K140M represent the K140 mother-child pair. The scale represents substitutions per site. (TIF) [file ppat.1004954.s003.tif]

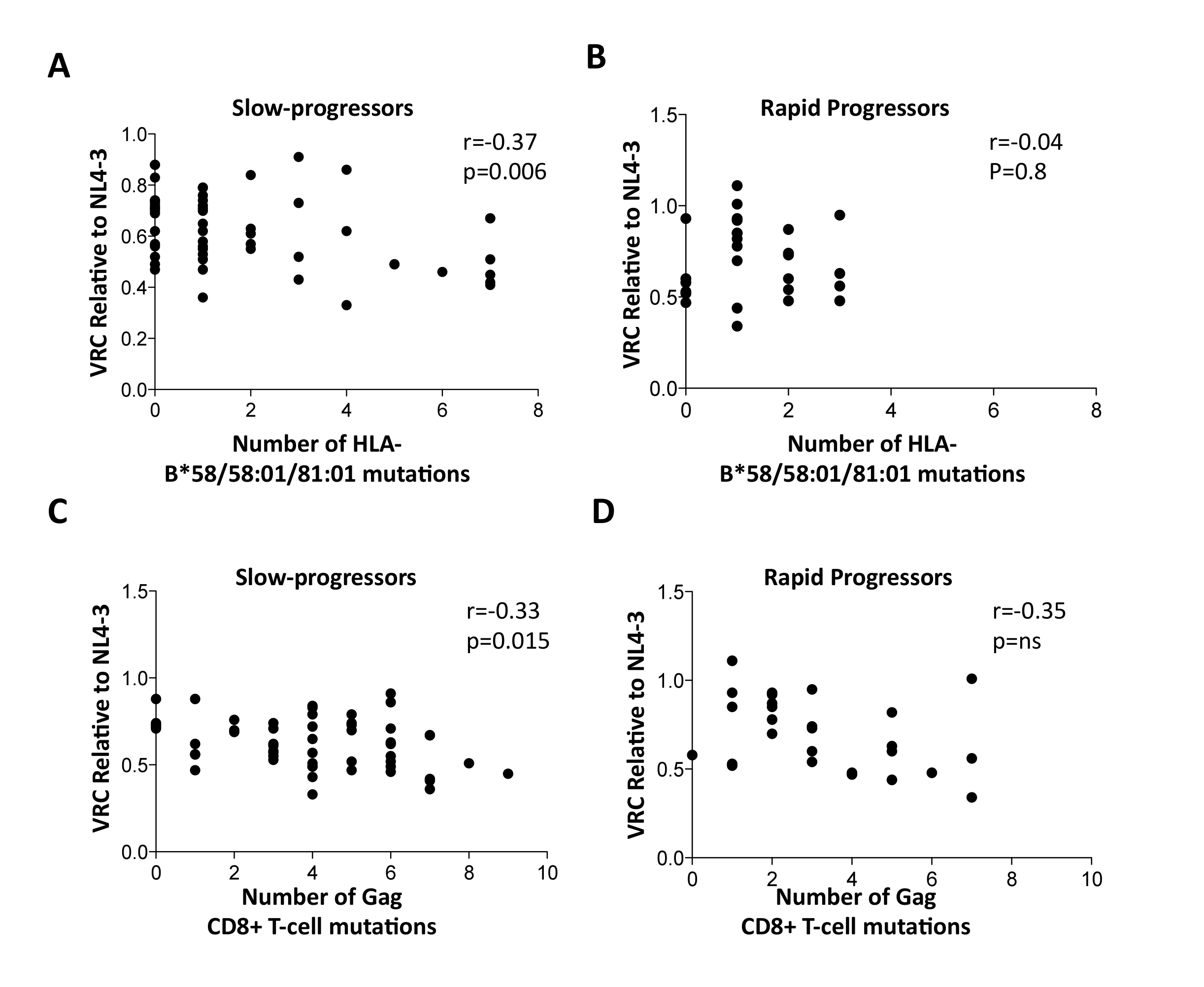

Supplement: S4 Fig — A-B: HLA-B*57/58:01/81:01-associated Gag mutants. A. Slow progressors. B. Rapid progressors. C-D. Gag-specific CD8+ T-cell mutations within defined epitopes (listed in S1 Table). A. Slow progressors B. Rapid progressors. (TIF) [file ppat.1004954.s004.tif]

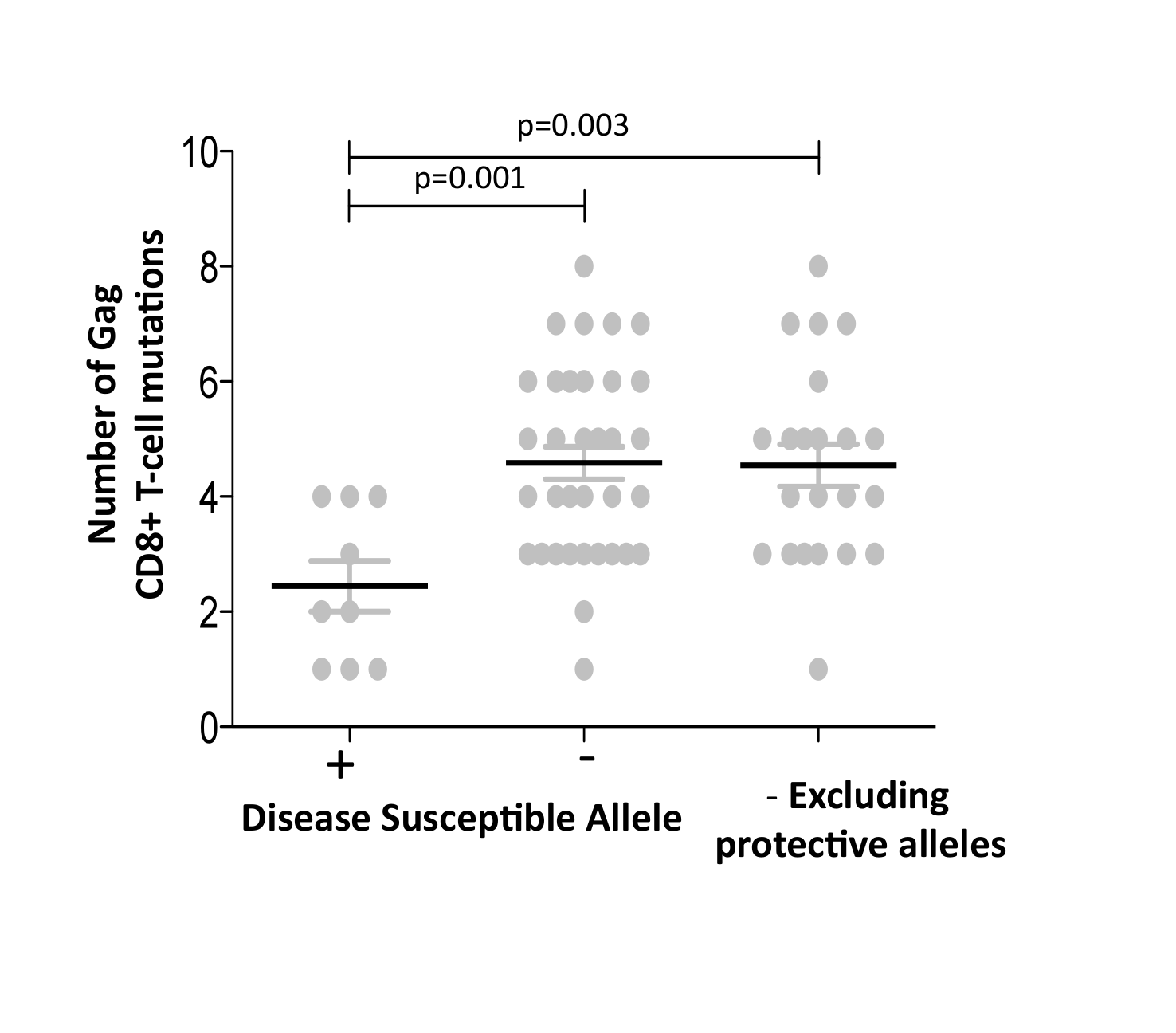

Supplement: S5 Fig — (TIF) [file ppat.1004954.s005.tif]

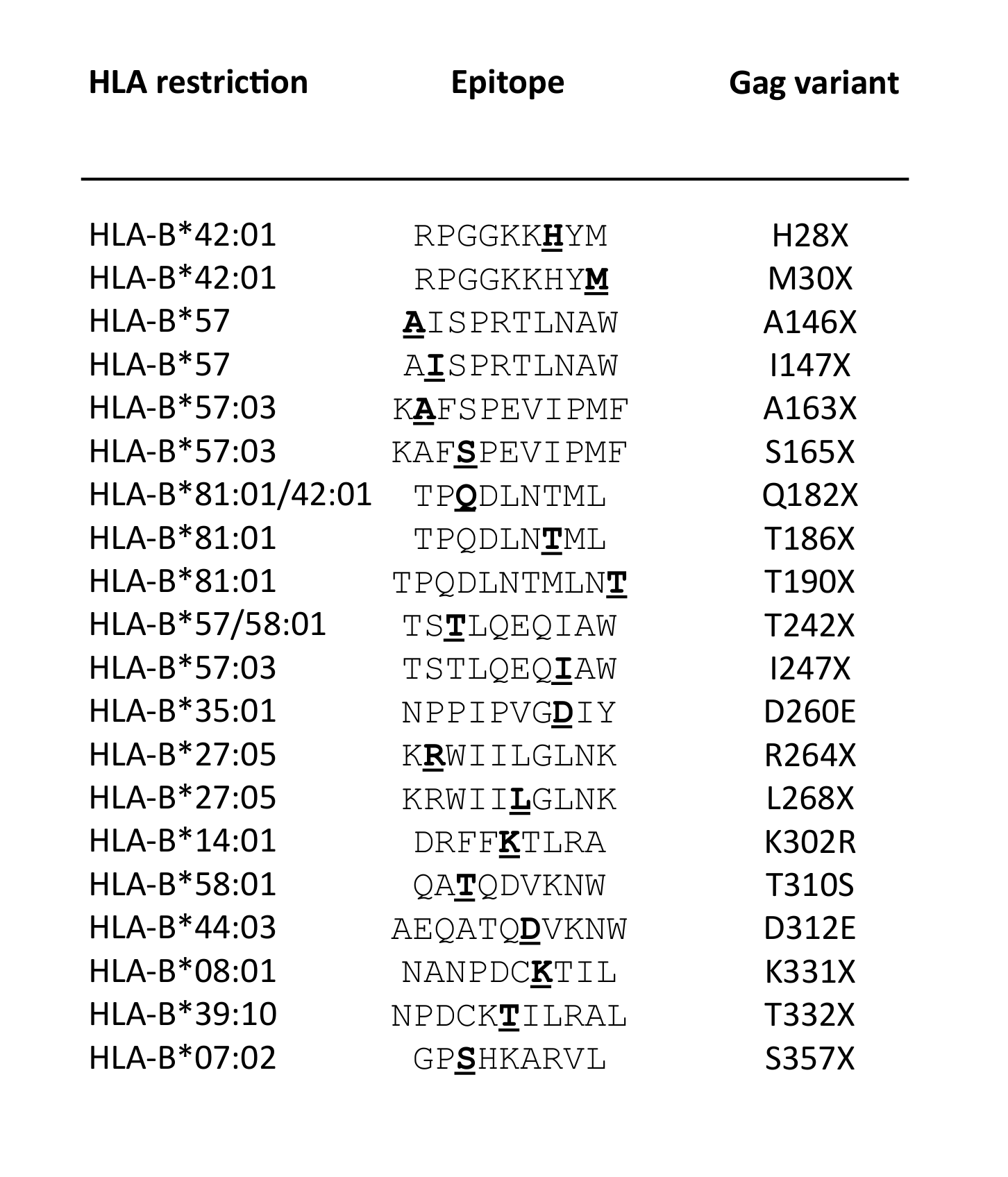

Supplement: S1 Table — (TIF) [file ppat.1004954.s006.tif]

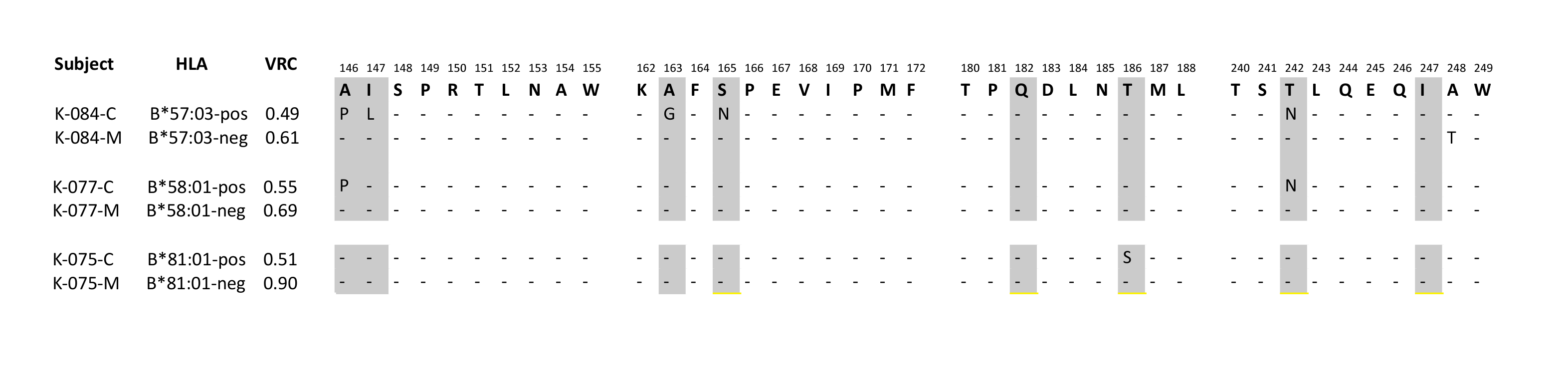

Supplement: S2 Table — Mutations shown; A146X and I147X within the HLA-B*57 restricted epitope ISPRTLNAW (ISW9, Gag 147–155); A163X and S165X within the HLA-B*57-restricted epitope KAFSPEVIPMF (KF11, Gag 162–172); Q182X and T186S within the HLA-B*81:01-restricted epitope TPQDLNTML (TL9, Gag 180–188); and T242X and I247X within the HLA-B*57/58:01-restricted epitope TSTLQEQIAW (TW10, Gag 240–249). (TIF) [file ppat.1004954.s007.tif]
